# First fluvial archive of the 8.2 and 7.6–7.3 ka events in North Africa (Charef River, High Plateaus, NE Morocco)

**DOI:** 10.1038/s41598-022-11353-y

**Published:** 2022-05-11

**Authors:** Bruno Depreux, Jean-François Berger, David Lefèvre, Quentin Wackenheim, Valérie Andrieu-Ponel, Sylvia Vinai, Jean-Philippe Degeai, Abderrahmane El Harradji, Larbi Boudad, Séverine Sanz-Laliberté, Kristell Michel, Nicole Limondin-Lozouet

**Affiliations:** 1 Univ. Paul Valéry Montpellier 3, CNRS, UMR 5140 Archéologie des sociétés Méditerranéennes, Campus Saint Charles, 34000 Montpellier, France; 2 LabEx Archimède, Univ. Paul Valéry Montpellier 3, Campus Saint Charles, 34000 Montpellier, France; 3 Univ. Lyon, Université Lumière Lyon 2, CNRS, UMR 5600 Environnement Ville Société, 69635 Lyon, France; 4 Univ. Paris 1, UPEC, CNRS, UMR 8591 Laboratoire de Géographie Physique, 92195 Meudon, France; 5 Univ. Paris 1, CNRS, UMR 8215 Trajectoires, 75004 Paris, France; 6 Institut Méditerranéen de Biodiversité et d’Ecologie Marine et Continentale, Aix Marseille Univ., CNRS, IRD, Technopôle de l’Environnement Arbois-Méditerranée, 13545 Aix-en-Provence, France; 7 Univ. Mohammed 1er, Oujda, Morocco; 8 Univ. Mohammed V, Rabat, Morocco; 9 Univ. Lyon, ENS de Lyon, CNRS, UMR 5600 Environnement Ville Société, 69342 Lyon, France

**Keywords:** Climate sciences, Hydrology

## Abstract

The Early–Mid Holocene transition is a period of profound changes in climatic mechanisms and hydrological features in Europe and North Africa. The melting of the Laurentide ice sheet led to an oceanic and atmospheric reorganisation in the North Atlantic, while the Mediterranean underwent a major hydrological shift. The impacts on Mediterranean rivers remain unclear, as there are few records documenting responses to the 8.2 ka event (the main Holocene climatic degradation). We present a fluvial record from Eastern Morocco documenting detailed hydrological variations from 8200 to 7500 cal. BP and their climatic forcing. A major hydrogeomorphic evolution of the Charef River occurred at that time, marked by two major incision stages close in time, under hyper-arid conditions at 8200 and ca. 7500 cal. BP. The impacts of these phenomena on the alluvial plains and associated archaeological records during Neolithisation, a major process in human history, currently remain unidentified. This new record sheds light on the fluvial response to the 8.2 ka event in North Africa and why other records are missing. We also bring new insights into the hydrological disruption at the Early–Mid Holocene transition, which was driven by the end of deglaciation combined with insolation and solar forcing. Furthermore, centennial solar variability may have paced river activity in the Moulouya basin and arid regions of North Africa.

## Introduction

The Early–Mid Holocene transition was a period of profound changes in climatic mechanisms and hydrological conditions in Europe and North Africa. During the Early Holocene, orbitally-induced warming caused the retreat of ice sheets^1^, notably melting of the Laurentide Ice Sheet (LIS), which was marked by sudden meltwater outbursts. Climate variability was therefore paced by millennial-scale solar irradiance changes and meltwater pulses, leading to Atlantic Meridional Overturning Circulation (AMOC) perturbations and the North Atlantic cold event^2,3^. The Early–Mid Holocene transition is marked by the final disappearance of the LIS and its last glacial lake outbursts: the major drainage of the Lake Agassiz-Obijway at 8470 cal. BP^4^ and a minor event at ca. 7500–7000 cal. BP^5^. These phenomena are thought to be the main factors behind the modification of oceanic and atmospheric circulations over the North Atlantic, which led to the emergence of a North Atlantic Oscillation (NAO)-like circulation pattern from the Mid-Holocene onwards in Europe and the Mediterranean^6,7^.

The drainage of the Lake Agassiz-Obijway probably led to the so-called 8.2 ka cooling event, which is the most well-known Holocene rapid climatic change (RCC), because it is the only one to be identified in the Greenland ice cores and numerous archives worldwide^2^. The hydrological impacts of the 8.2 ka event varied across Europe and the Mediterranean basin, with a latitudinal partition of hydrological responses to long- and short-term climatic changes evidenced during the Holocene^6^. The 8.2 ka event resulted in wetter and colder conditions between ~ 50° to ~ 42°N, and drier conditions north and south of these latitudes^8^. These impacts are well evidenced in marine^9–11^, lacustrine^12^, and speleothem records^13,14^, although surprisingly, the river responses to the 8.2 ka event are actually rather unknown in Western Europe and the Mediterranean^15,16^. The end of the 8th millennium BP (ca. 8500–8000 cal. BP) is marked by high river stability in the UK^17^, and conversely, by high river activity in Germany^8^. In the Mediterranean, the most comprehensive fluvial synthesis indicates that the 8600–7800 cal. BP interval was marked by a common absence of flooding in southern France, southern Italy, the eastern Mediterranean, Iberian Peninsula, Tunisia, and Morocco^16^. This is less significant for Iberia, as it is mainly based on slack water deposits which cannot be correlated with alluvial plain records. A few detailed studies from the northern Mediterranean shed light on the fluvial dynamics at that time. Two high-resolution palaeochannel records from northern Greece and southern France demonstrate overall high fluvial activity related to the 8.2 ka event, marked by a succession of coarse deposits, with braiding and fluvio-colluvial aggradation, and pedogenic development, respectively, in the following centuries^18,19^. A coarse-load flood deposit that eroded part of the Early Neolithic Khirokitia archaeological site and led to spatial redistribution of the village is also noted in Cyprus^19^. The most detailed record, located on an Early Holocene terrace in the apex of the Holocene alluvial fan of the Citelle River in the Middle Rhone valley, shows overall increased fluvial activity over a millennium (8200–7100 cal. BP), before an entrenchment after 7000 cal. BP, due to wetter climatic conditions related to the 8.2 and 7.6–7.3 ka RCCs^18^. This archive mirrors the variations recorded by the Corchia and Renella speleothems from north-central Italy, which indicate increased winter precipitation and torrential flooding in mountainous regions between 8200 and 7100 cal. BP^20^. In Spain, few detailed regional case studies present alluvial records for that time, indicating regional floodplain aggradation around 8000–7300 cal. BP^21^. This refers to a recent study on the Galera River which allows the construction of a coherent regional palaeoenvironmental scheme for SE Spain indicating particularly wet conditions between 8000–7500 cal. BP, interrupted by dry episodes^22^. Regarding depositional settings, there is a notable lack of Holocene flooding dates coming from palaeochannels^15^. Tunisian alluvial syntheses also provide no evidence of any fluvial response to the 8.2 ka event, mirroring the long-unresolved “missing event” in the SW Mediterranean^23^. As for Iberian records, it is interesting to see that Early Holocene deposits as a whole are particularly underrepresented^21,24^, which may be partly a problem of dating strategies. In Morocco, the Moulouya upstream rivers showed extensive wetland formation during the Early Holocene, whereas downstream archives do not record alluvial deposits at that time^25^. The common sedimentary gap spanning from 8700 to 7300 cal. BP in the NE Moroccan fluvial records is indicative of the sedimentary “silence” that characterises the Early–Mid Holocene transition in the Mediterranean rivers.

**Figure 1:**
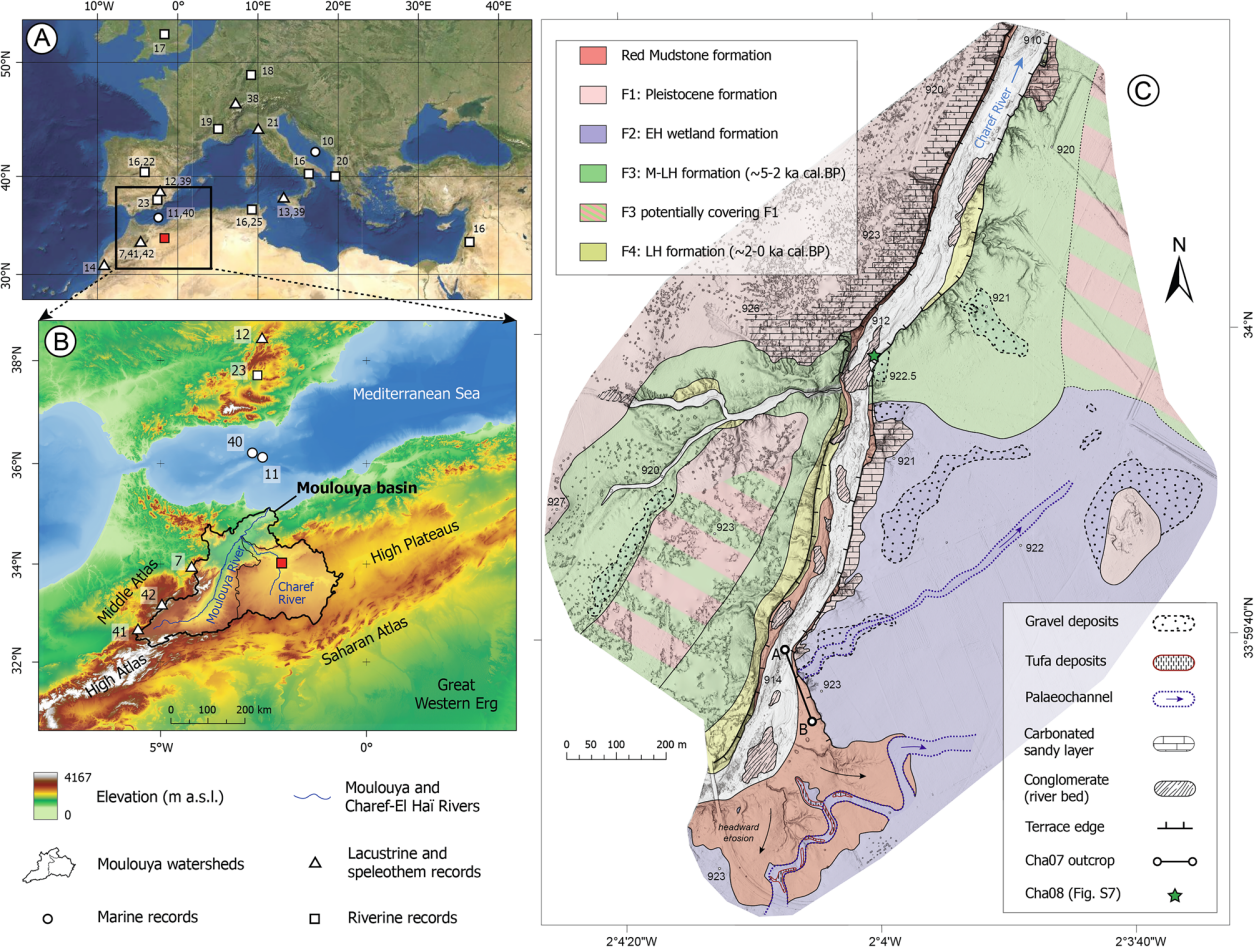
Geographical setting. (**A**) Location of the studied area and the references discussed in the Mediterranean basin (the numbers refer to the studies referenced in the text), and (**B**) within the topographic frame of NW Africa. (**C**) Geomorphological map of the superficial sedimentary formations of the studied section of the Charef River. *EH* Early Holocene, *MH* Mid-Holocene, *LH* Late Holocene. Data source and software: (**A**) data from TerraColor imagery (https://www.arcgis.com/home/item.html?id=10df2279f9684e4a9f6a7f08febac2a9), (**B**) data from ASTER GDEM2 (https://lpdaac.usgs.gov/products/astgtmv002/) both plotted by means of ArcGIS Pro [2.7] (https://www.esri.com/en-us/arcgis/products/arcgis-pro/), (**C**) map created by B. Depreux using Adobe Illustrator CC (https://www.adobe.com/).

However, the new record from the Charef River in the North African High Plateaus (NE Morocco) revealed in this paper sheds light on this issue. We present high-resolution chronostratigraphic and palaeoecological analyses of this fluvial sequence from the main tributary of the Moulouya River, one of the largest Mediterranean river basins (Fig. 1A). This record, spanning from 8200 to 7500 cal. BP, also brings new insights into the hydrological disruption at the Early–Mid Holocene transition in the western Mediterranean.

## Results

### Geomorphology, sedimentology, and chronostratigraphy

**Figure 2:**
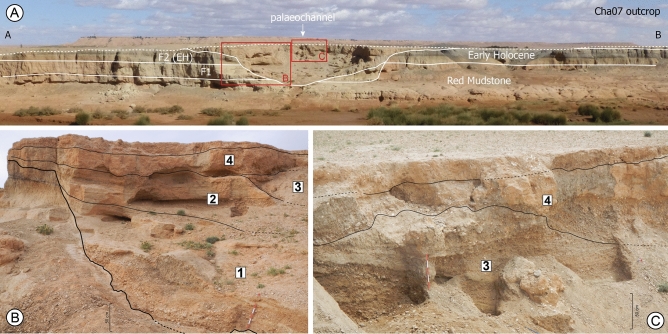
(**A**) Overview of the Cha07 outcrop, showing the Cha07 palaeochannel record and its incision into F2, F1, and the Red Mudstone formation. (**B**,**C**) Views of the palaeochannel infilling showing the limits and numbers of the fluvial sequences. The palaeochannel does not present a straight outcrop but has been hollowed out by erosion and has a platform at the top of the sequence 1. Data source: (**A**–**C**) pictures from D. Lefèvre, J.-F. Berger, and B. Depreux, respectively.

The Cha07 fluvial record corresponds to a palaeochannel infilling located in the riverbanks of the Charef River, the main tributary of the Moulouya River, which drains the current arid High Plateaus region and the Ain Beni Mathar basin (Fig. 1B, Supplementary Fig. S1). The river section, intensively surveyed over a 2-km upstream–downstream section, shows several nested alluvial formations spanning the Holocene (Fig. 1C). Fine wetland deposits (F2) accumulated in the southeast part of the river section, and are developing eastwards, whereas there is an underlying carbonated massive sands (F1) outcrop in the northwest part (Fig. 1C). F2 corresponds to the climate-driven extensive Early Holocene formation recognised in the upper and middle reaches of the Moulouya catchment^25^. Owing to the current entrenchment of the river bed, a palaeochannel (Cha07 outcrop) cutting into F1, F2, and the Red Mudstone formation was identified (Fig. 2A). More details about the different Holocene formations identified in the area are given in the Supplementary Information.

**Figure 3:**
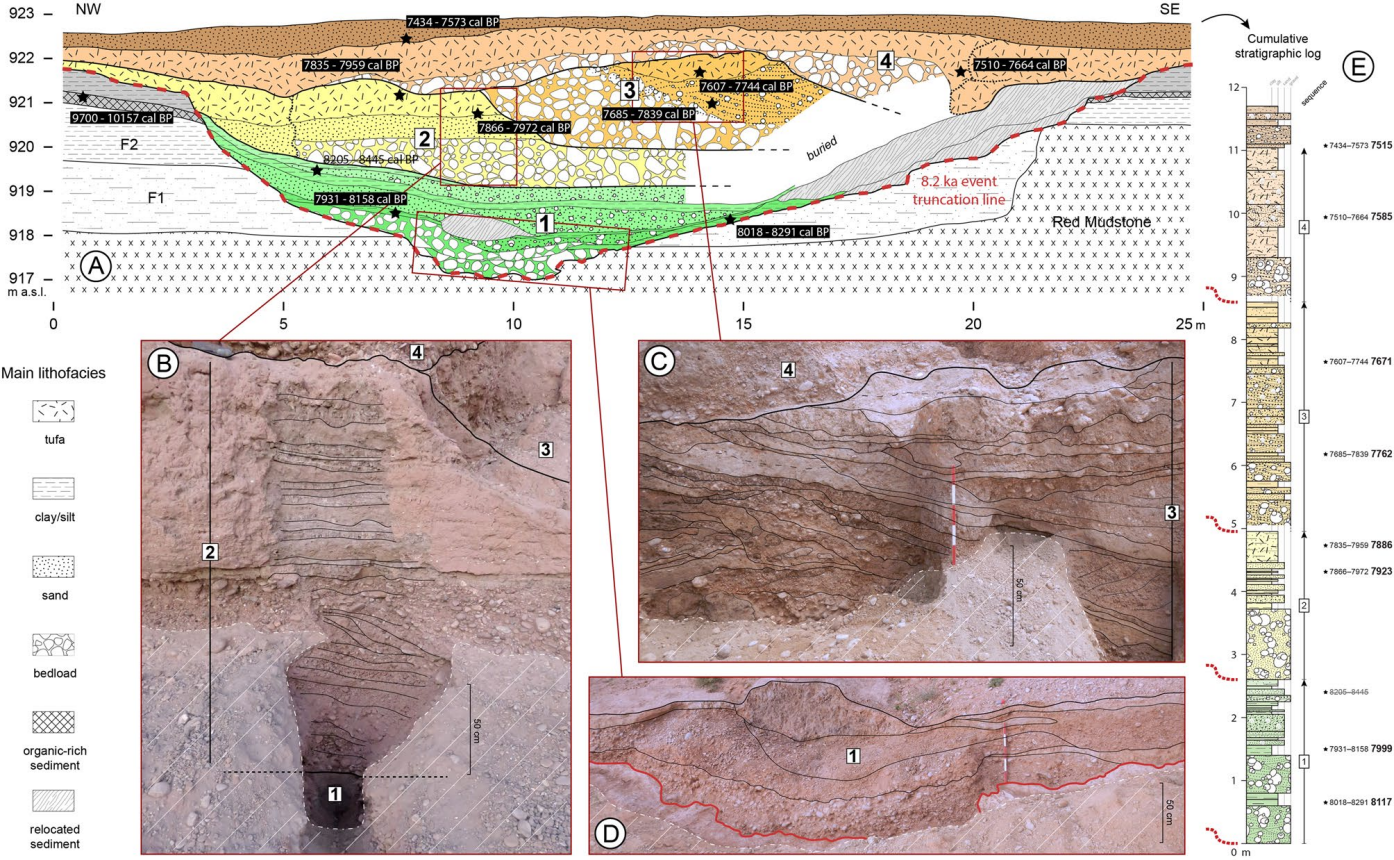
(**A**) Transverse section across the Cha07 palaeochannel infilling showing the main litho-stratigraphic units, positions of radiocarbon datings (2σ cal. BP), and underlying formations: Early Holocene F2, pre-Holocene F1, and Red Mudstone formation. (**B**–**D**) Detailed views of parts of the palaeochannel infilling with stratigraphic units. (**E**) Cumulative stratigraphic log with litho-sedimentary units grouped into fluvial sequences. Data source and software: drawing by B. Depreux using Adode Illustrator CC (https://www.adobe.com/), and pictures from B. Depreux and J.-F. Berger.

**Figure 4:**
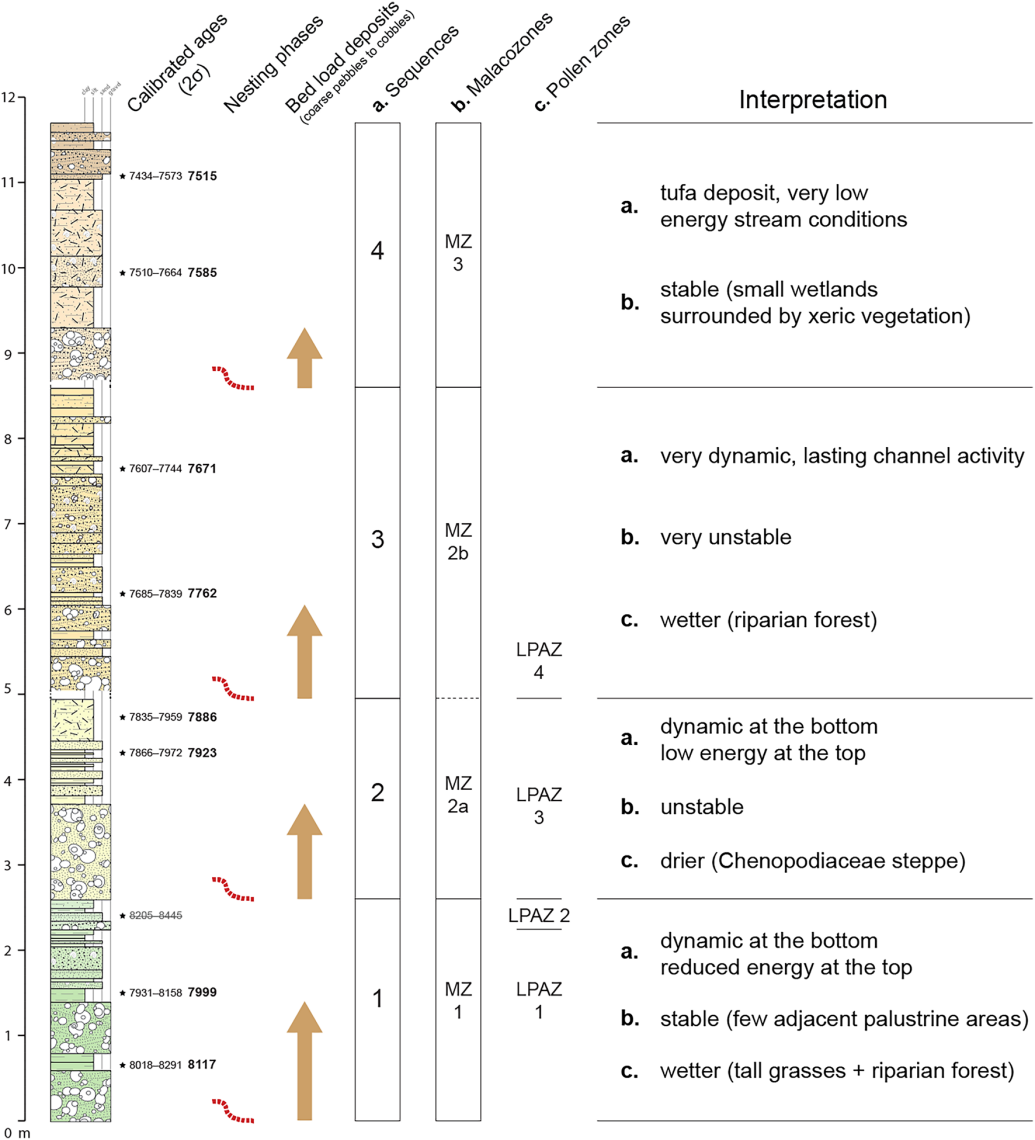
Synthetic results from the stratigraphic, sedimentological, malacological, and pollen analyses of the Cha07 palaeochannel infilling.

The 18-m-wide palaeochannel shows a 6-m-thick infilling composed of alternating coarse and fine sediments (Fig. 3). The time span encompassed by the Cha07 record is constrained by eight radiocarbon dates ranging from 8150 to 7500 cal. BP (Supplementary Table S1). A rapid succession of aggradation and small incision phases is evidenced by several nested channel deposits, implying strong energy variations of the active channel and strong lateral and vertical mobility (Fig. 2B,C). This channel shows a polyphase activity with four different cycles according to morphology, incisional features, and sedimentology, and will be described hereafter as fluvial sequences (Figs. 3 and 4). All the sequences are nested into the previous deposits, and present sedimentary graded bedding with channel bed load deposits at the bottom (coarse pebbles to cobbles), grading upwards into finer particles (silt and clay). This upwards-fining trend indicates a progressive decrease in flow energy within a channel deposit. These fluvial sequences indicate four periods of aggradation dated around 8116–7999 (S-1), 7923–7886 (S-2), 7762–7671 (S-3) and 7585–7515 cal. BP (S-4), which are separated by erosive phases of the previous deposit over 1.5–2 m . No such fluvial archive was found in North Africa, which makes this record a rare opportunity to document hydrological and climatic changes during the Early–Mid Holocene transition. Indeed, the chronology of this archive perfectly fills a sedimentary hiatus generalised to the whole alluvial archives of NE Morocco, which extends from 8700 to 7300 cal. BP^25^ (Supplementary Fig. S6). This lack of alluvial records has long prevented an understanding of the response of hydrosystems to the 8.2 ka climatic event, which remained totally unknown^23^.

The incision I-1 of the Cha07 palaeochannel into F2 and F1 down to the Red Mudstone formation indicates a major downcutting phase ca. 8200 cal. BP of at least 4 m as organic-rich fluvio-palustrine deposits suggest a water table level at 921 m a.s.l. (Fig. 3A,D). Indeed, the top of what is preserved of the Early Holocene F2 deposits, strongly eroded by the palaeochannel Cha07, was dated to 9700–10,157 cal. BP. These F2 deposits correspond to the regional-scale wetland formation, documented within the Tabouda River, the main tributary of the Charef River, and in the Ksabi basin in the Middle Moulouya (Supplementary Fig. S1). The study of these alluvial deposits, highlighting the similarities and synchronies of fluvial dynamics and topogeomorphological positions between these two basins, which are more than 300 km apart, has shown a strong homogeneity of hydrosedimentary dynamics and morphogenesis at the regional scale. This wetland formation, which we assume to reflect the orbitally-induced African Humid Period (AHP), demonstrates the high sensitivity of these catchments and North African highlands to climatic forcing^25^. In all the deposits studied, and in both basins, this formation is truncated. The most recent date was obtained from the Tab01 outcrop, not far from the Charef River (Supplementary Fig. S1), and confirms the development of this formation until at least 8910 cal. BP^25^.

Following I-1, the first sequence (S-1) exhibits a 1-m-thick bed load deposit defined by nested coarse pebbles (1.6–6.4 cm) to cobbles (6.4–25.6 cm) interstratified with laminated silty beds and dismantled Early Holocene sediments (Supplementary Fig. S8). The channel deposit ends with alternating sands and granules to clayey units. S-2 presents a 1-m-thick homogeneous and monogenetic bed load deposit, then well-sorted horizontal sandy and silty beds (Fig. 3B). This channel infilling ends with tufaceous sandy silts that include complete *Potomida littoralis* bivalves. In both sequences, several polyhedral clayey beds marked by desiccation cracks indicate episodes of drying up with an absence of discharge. Sequences 3 and 4 present slightly different sedimentary features. S-3 shows a 2-m-thick gravel aggradation interstratified with sandy and silty beds marked by the presence of reworked tufa tubes and rolled clay pebbles. Then, a lateral accretion and displacement of the channel towards the southeast, characterised by cross-bedded sands, indicates more lasting channel activity between 7800 and 7675 cal. BP (Fig. 3C). This sequence ends with a 60-cm-thick tufa deposit including numerous tubes and complete *Potomida littoralis*. Above its bedload deposit, S-4 does not exhibit rhythmic sand and silt layers, only a 1.7-m-thick tufa sedimentation reflecting reduced discharge and stabilisation at the basin scale, as for the top of S-2 and S-3. The last deposit dated to 7500 cal. BP, which covers the top of the palaeochannel with a poorly sorted layer (gravel to silty fractions), does not provide evidence of channel formation, but a mixed flood deposit implying that the channel has migrated.

**Figure 5:**
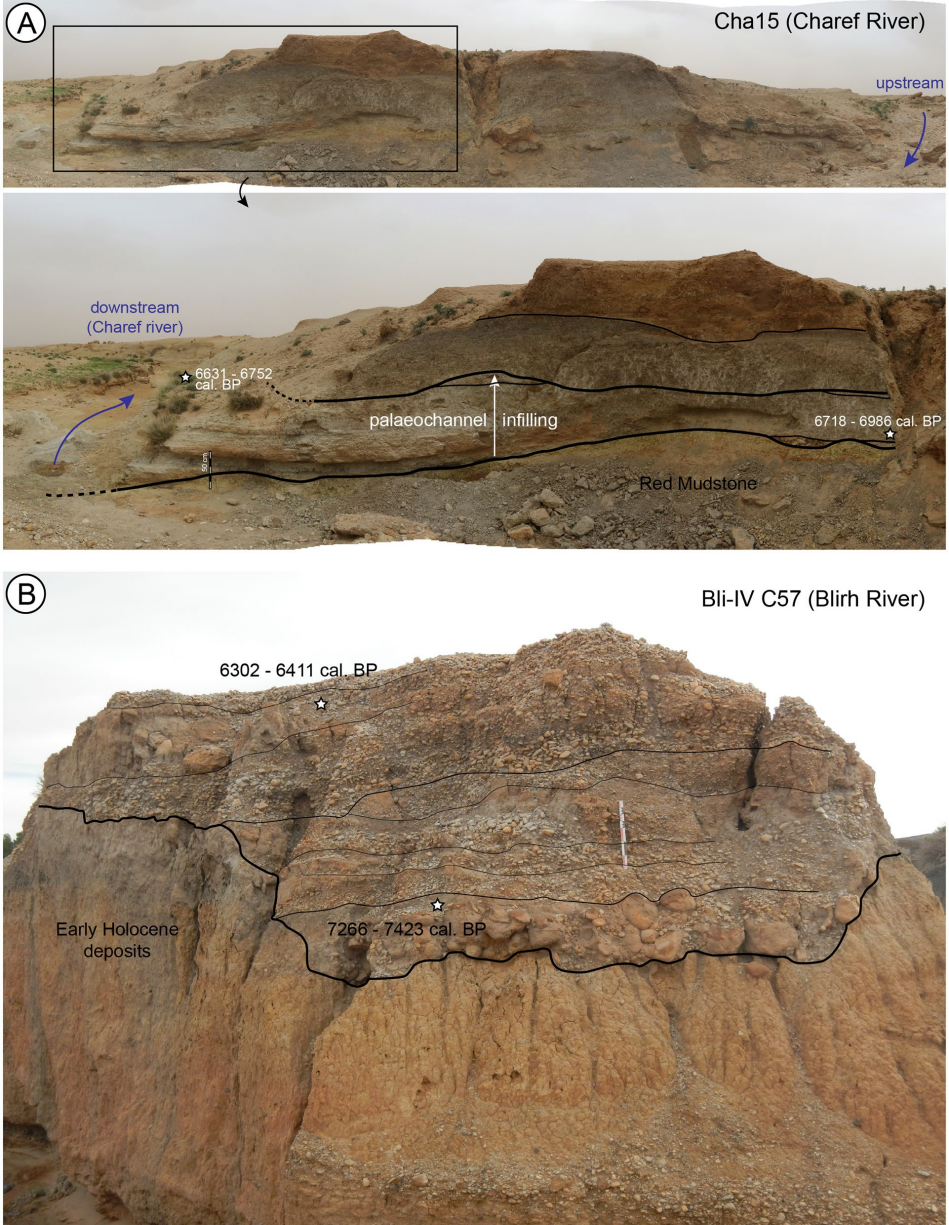
Views of the two other partially eroded palaeochannels identified in the Moulouya's upstream basins. (**A**) The Cha15 outcrop located 1.5 km upstream of Cha07, on a small tributary 10 km long of the Charef River, (**B**) the Bli-IV C57 outcrop located in the Blirh River in the Ksabi basin (middle Moulouya).

Apart from the Cha07 palaeochannel studied here, only two other palaeochannels have been identified in the upstream basins of the Moulouya. The first is located 1.5 km upstream of Cha07, on a small tributary 10 km long of the Charef River (Supplementary Fig. S1). The second is located in the Blirh River in the middle Moulouya, 300 km away. Both records show a similar pattern of incision into the Early Holocene formation and the Red Mudstone formation. In contrast to Cha07, the entire palaeochannel section is not preserved, but only one of the lateral edges in a proximal position. The first (Bli-IV C57) shows the succession of bedload and travertine silt deposits between 7350 and 6350 cal BP (Fig. 5B). The incision thus slightly pre-dates 7350 cal. BP. The second (Cha15) is less well dated, showing a silty-sandy fill, sometimes tufaceous and rich in malacofauna (Fig. 5A). Two dates were made at two different locations in the centre of its fill and give ages between ca. 7000 and 6650 cal. BP. These data show a regional incision phase before 7350 cal. BP, and thus confirm firstly the existence of the I-2 incision between 7500 and 7400 cal. BP, as assumed by the Cha07 archive, and secondly, the role of external climatic forcing on the avulsion and palaeochannel formation processes.

These signatures are indicative of a rapid geomorphological evolution of the Charef floodplain occurring after a multi-millennial period of stability (the Early Holocene wetland formation^25^). The channel trajectory at the western edge of the Early Holocene alluvial plain, marked by a SW–NE palaeocourse, evidences a river migration towards the northwest. This migration was not progressive, but rather involved channel avulsion due to very high discharge increases and fast channel aggradation. This north-westward migration appears to have continued as evidenced by Mid to Late Holocene deposits (F3–F4), including channel deposits at the base of F3 along the present valley axis (Supplementary Fig. S2), and the present course of the Charef River (Fig. 1C).

### Palaeoecology

The malacofauna of the Cha07 record comprises a high proportion and diversity of freshwater molluscs and fewer terrestrial snails, which reflects the local environmental conditions of the channel bottom and the river bank. The molluscan succession can be divided into three biozones (MZ1, MZ2a&b, MZ3) based on ecological patterns (Supplementary Fig. S9) and the occurrence and development of taxa (Supplementary Fig. S10). The assemblages from Malacozone 1 indicate a dominantly aquatic environment with few adjacent palustrine areas and sparse xeric vegetation. From 7950 cal. BP onwards, Malacozone 2 records strong fluvial dynamics that affect the sustainability of the terrestrial environments. The stability of the terrestrial environments suggests the development of wetland areas near the channel whereas dynamic fluvial conditions would prevent the establishment of adjacent marshy habitats. From 7850 to 7700 cal. BP, habitat instability is particularly pronounced, and corresponds to the sub-zone MZ2b. Molluscan assemblages in Malacozone 3 reflect the predominance of aquatic environments. However, after 7700 cal. BP, the adjacent riverbanks stabilise and small wetlands develop surrounded by xeric vegetation in the channel zone. The palaeoenvironmental scheme obtained from mollusc assemblages supports the lithostratigraphical data.

The vegetation patterns evidenced by the pollen analysis indicate a sparsely wooded landscape dominated by a steppe of Poaceae, sagebrush, and Chenopodiaceae (Supplementary Fig. S11). At the bottom of the record, perennial water bodies in the bed of the Charef River allow the existence of a population of *Myriophyllum*, the presence of tall grasses (*Sparganium/Typha*) along the banks, and a riparian forest dominated by *Salix*. This environment was propitious for herds of large herbivores to come and drink. The climate then became drier, as indicated by the domination of Chenopodiaceae steppe over the *Artemisia* and Poaceae steppe that previously prevailed. The disappearance of the riparian forest is consistent with the aridification of the climate. This event is dated between 7866–7972 and 7931–8158 cal. BP. At the top of the pollen record, the climate becomes wetter, as indicated by the recovery of *Artemisia* and Poaceae steppe and a new phase of colonisation of the river banks by a riparian *Tamarix* forest. Fire signals dated to between 7835 and 7972 cal. BP could be either of natural or anthropogenic origin. The question of agricultural signals arises because pollen of cereals and nitrophilous plants and spores of coprophilous fungi have been recorded for dates between 7931–8158 and 8018–8291 cal. BP. These could be fleeting signs of human occupation and the presence of grazing animals, not necessarily domesticated; it is possible the appearance of cereals is natural and results from biotic interactions between the steppe and large wild herbivores, without human involvement being necessary, as has been shown in Turkey^26^. Hunter-gatherer populations and herds of large mammals may have benefited from the potentially edible plants present in the vegetation (Supplementary Fig. S11), particularly cereals, which are rich in carbohydrates and very nutritious.

## Discussion

### The missing event: the 8.2 ka event in the Mediterranean and arid North African rivers

While drier conditions seemed to result in higher geomorphic stability in northern European rivers^17,27^, what impact did the 8.2 ka RCC have on river responses in the southern Mediterranean and (semi-)arid North Africa? How do we explain the Mediterranean-scale paucity of fluvial deposits for this time?

In the upstream Moulouya basin, no deposits apart from the Cha07 palaeochannel record have been dated to this period, regardless of the sedimentary facies (channel bed, overbank, hydromorphic, or pedogenic sediments). This sedimentary gap also concerns the entire alluvial record of NE Morocco, which extends from 8700 to 7300 cal. BP^25^, and North Africa as a whole^23^. We can therefore question why such intense fluvial activity does not leave any flood deposits preserved in the floodplains. In this study, we assume that the 8.2 ka RCC had a major hydrological impact in North Africa, marked by increased river activity, and that taphonomic conditions are a key to understanding the sedimentary hiatus at that time.

**Figure 6:**
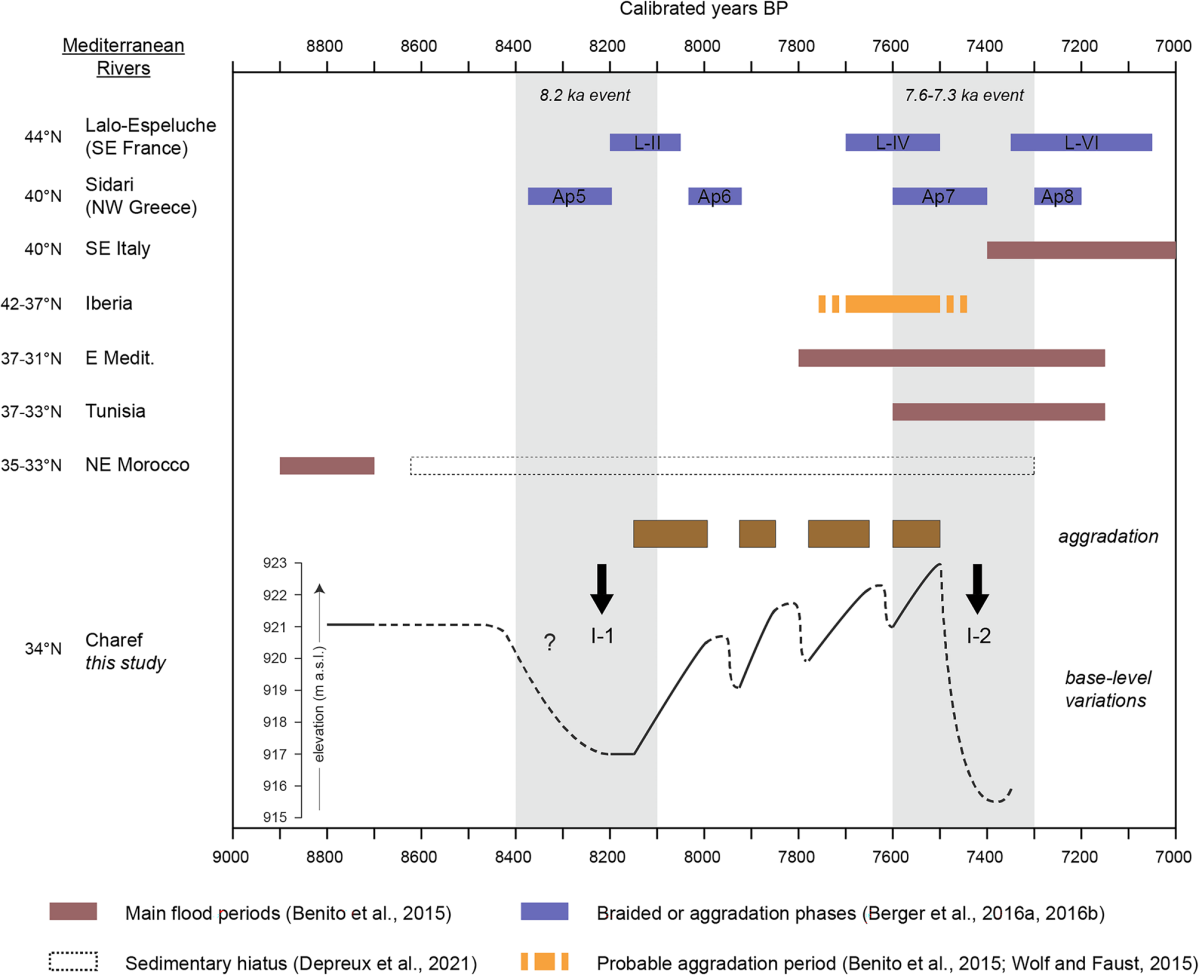
A synthesis of river activity in the Mediterranean at the Early–Mid-Holocene transition, including the main flood periods in SE Italy, Iberia, eastern Mediterranean, Tunisia, and Morocco^16^, the sedimentary gap in NE Moroccan rivers from 8700 to 7300 cal. BP^25^, indices of aggradation in Iberia^21^, the braided phases identified at Lalo-Espeluche^18^, the aggradation phases identified at Sidari^19^, and the base-level variations of the Charef River with the I-1 and I-2 incision phases and the four aggradation phases.

Climate forcing is indeed the most likely cause because of the good correlation with the regional palaeoclimatic record, discussed below, and the regional character of the observed changes. Furthermore, the role of other factors is not convincing. Archaeological data is almost non-existent in the region, apart from a few rare and isolated studies. Human settlement is therefore unknown in the upstream basins of the Moulouya for this period^28^. Even on the Mediterranean coast, the period corresponds to a hiatus in the archaeological sequences at the Epipaleolithic-Neolithic transition^29^. On the basis of pre-existing data, it is difficult to imply that the anthropogenic factor is responsible for hydrogeomorphological dynamics representative of the upstream basins. Regarding tectonics, the High Plateaus, belonging to the Eastern Mesetian domain, are not affected by either the Atlasic orogeny (Jurassic and Eocretaceous in age) or the Cenozoic Rifan orogeny^30^. No evidence of neotectonics has been identified in the Holocene (or older) sedimentary formations, so it does not appear to be active during this period. Thus, the lack of fluvial responses to the 8.2 ka RCC in the southern Mediterranean and concurrent with the exceptional hydrological conditions recorded in the Charef River are not down to mere coincidence. This assessment is supported by other aforementioned detailed channel records from the northern Mediterranean^18,19^ (Fig. 6).

The Cha07 record demonstrates that the drier conditions of the 8.2 ka RCC, followed by wetter conditions, resulted in strong hydrosedimentary dynamics marked by severe river incision (I-1), high-flow discharge, and coarse bedload transport and deposition. Indeed, fluvial dynamics in (semi-)arid environments respond in a different way to precipitation fluctuations, and river incision, when climate forcing is involved, appears to be caused more by high-energy discharges and by a transition from arid to wet conditions^31^. For the Charef River, incision accompanied by coarse fluvial deposits indicates highly unstable landscape conditions and strong liquid discharges that may reflect the transition from drier to wetter conditions. A common pattern is that arid conditions reduce vegetation cover and soil infiltration capacity, leading to increased soil erosion and surface runoff^32^. A Horton-type overland flow characterised by an excess amount of water that bare soil cannot absorb is common in such environments where precipitation patterns favour scarce but intense rainfall. Indeed, NW Africa, excluding Atlantic Morocco, is subject to spring to summer precipitations due to cyclogenesis and convective storms^33^. This pattern explains the general trends in floodplain sediment aggradation in (semi-)arid river systems in relation to drier conditions^21^.

These strong hydrosedimentary dynamics led to the erosion and removal of previous alluvial deposits, as evidenced by the common sedimentary gap spanning from 8700 to 7300 cal. BP in the NE Moroccan fluvial records^25^. Moreover, the first evidence of sedimentation after the last Cha07 channel (S-4) is a channel deposit of F3 dated to 4855–5231 cal. BP (Cha08 outcrop) located 700 m downstream and incising the Red Mudstone formation below the present base level (913 m a.s.l., Supplementary Figs. S4 and S7). The difference in elevation between the last channel deposits of Cha07 (S4, dated ca. 7600 cal. BP) and Cha08 (dated ca. 5000 cal. BP) is 8 m (Supplementary Figs. S3 and S4). If the present slope of the thalweg (− 1 m) is removed, a drop in base level of 7 m is recorded between 7600 and 5000 cal. BP. This sedimentary hiatus in the Charef River from 7500 to ca. 5000 cal. BP thus reveals a long-term entrenchment of the river bed, which probably contributed to post-depositional erosion of alluvial deposits related to the 8.2 or 7.6–7.3 ka events. This has partially preserved the F2 formation, as it is largely developed within the alluvial plain, while the alluvial deposits related to the 8.2 and 7.6–7.3 ka events, of a different nature (palaeochannels), and in a proximal position in the axis of the valley, have been eroded by incision accompanied by vertical and lateral bank erosion. We therefore postulate that the lack of alluvial deposits in North Africa ca. 8200 cal. BP^23^ stems from strong post-depositional erosion and the predominance of coarse fluvial deposits resulting from increased channel dynamics and erosional processes. Palaeochannels, as an alluvial archive, are underrepresented in such environments, especially as they are located in the river stream and are therefore often completely removed by later erosional processes.

Besides alluvial deposits, these taphonomic conditions have impacted the preservation of archaeological remains. Recent probability distributions of ^14^C dates from archaeological sites, interpreted as demographic indicators, show a gap in archaeological deposits between 8600 and 7600 cal. BP. Explanations for this gap include a collapse in continental sites with a moving to coastal areas within the Eastern Rif–Lower Moulouya area^29^, and a decrease in human population with two significant peaks at 8400 and 8100 cal. BP at a Moroccan scale^34^. With mobility being one of the adaptive abilities of societies and being probable in such contrasting geographical settings forming a mosaic of environments, taphonomic processes should be taken into account, as floodplain open-air sites would have been eroded and removed during this period of fluvial instability^35^. We believe that interpretations of the amount of archaeological radiocarbon dates as human population decreases should be qualified by considerations of local settlement relocation and sedimentary taphonomic effects.

### Hydroclimatic shifts during the 8th millennium: forcing and taphonomic effects

#### A long-term fluvial instability at the EH–MH transition

**Figure 7:**
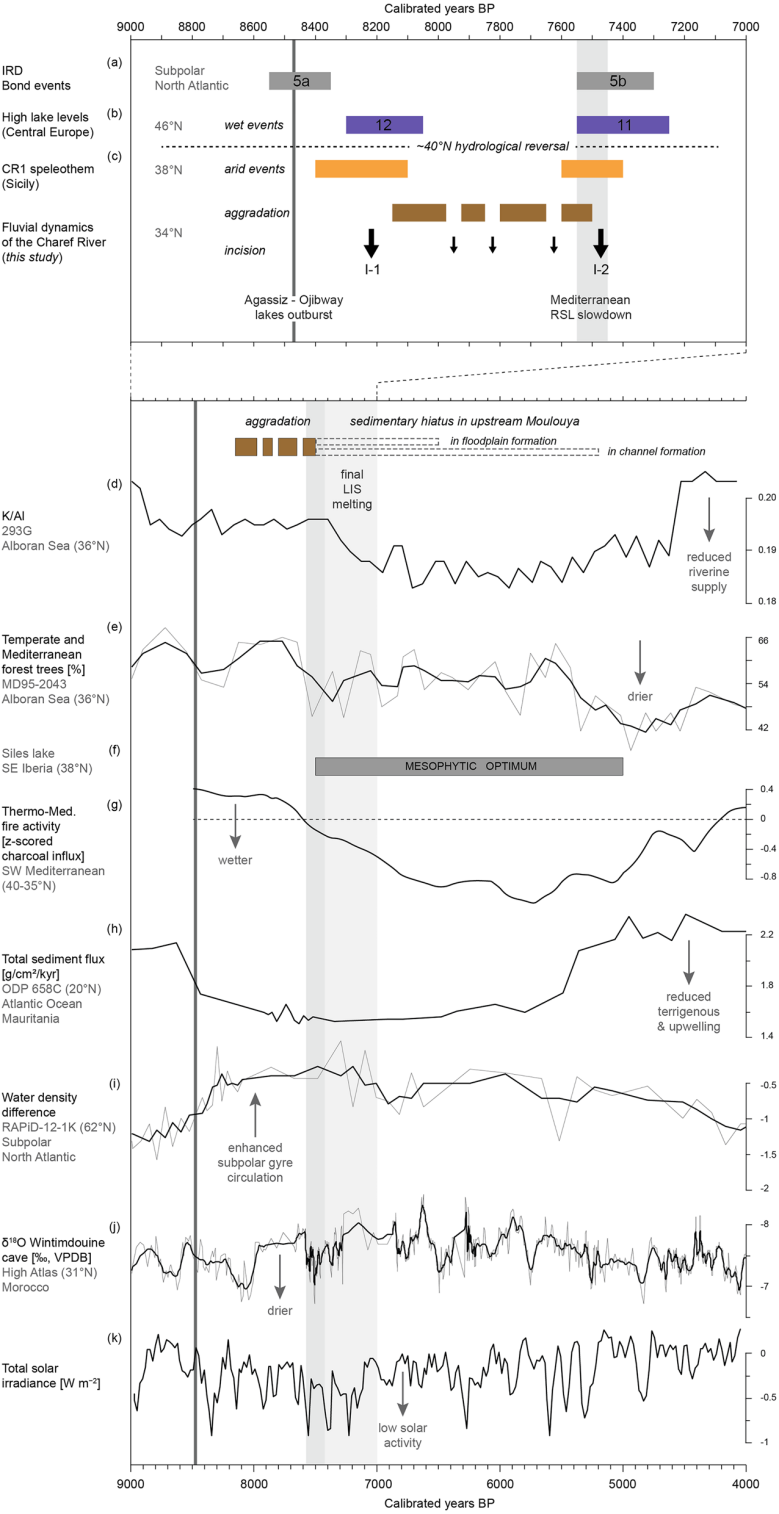
Palaeoenvironmental and palaeoclimatic proxies. (a) Stacked ice-rafted debris events^3^, (b) mid-European high lake levels^38^, (c) δ^18^O depletion from the CR1 speleothem record at the Carburangeli cave in Sicily^13^, (d) K/Al ratio at the 293G site in the Alboran Sea^40^, (e) temperate and Mediterranean forest trees at the MD95-2043 site in the Alboran Sea^11^, (f) mesophytic optimum at the Siles lake in SE Iberia^12^, (g) thermo-Mediterranean fire activity in SW Mediterranean^39^, (h) total sediment flux at the ODP 658C site off Mauritania^46^, (i) water density difference at the RAPID-12-K site in the subpolar North Atlantic^44^, (j) δ^18^O from the speleothem record at the Wintimdouine cave in Morocco^14^, (k) total solar insolation (TSI, W m^−2^) derived from ^14^C and ^10^Be radionuclides^37^.

**Figure 8:**
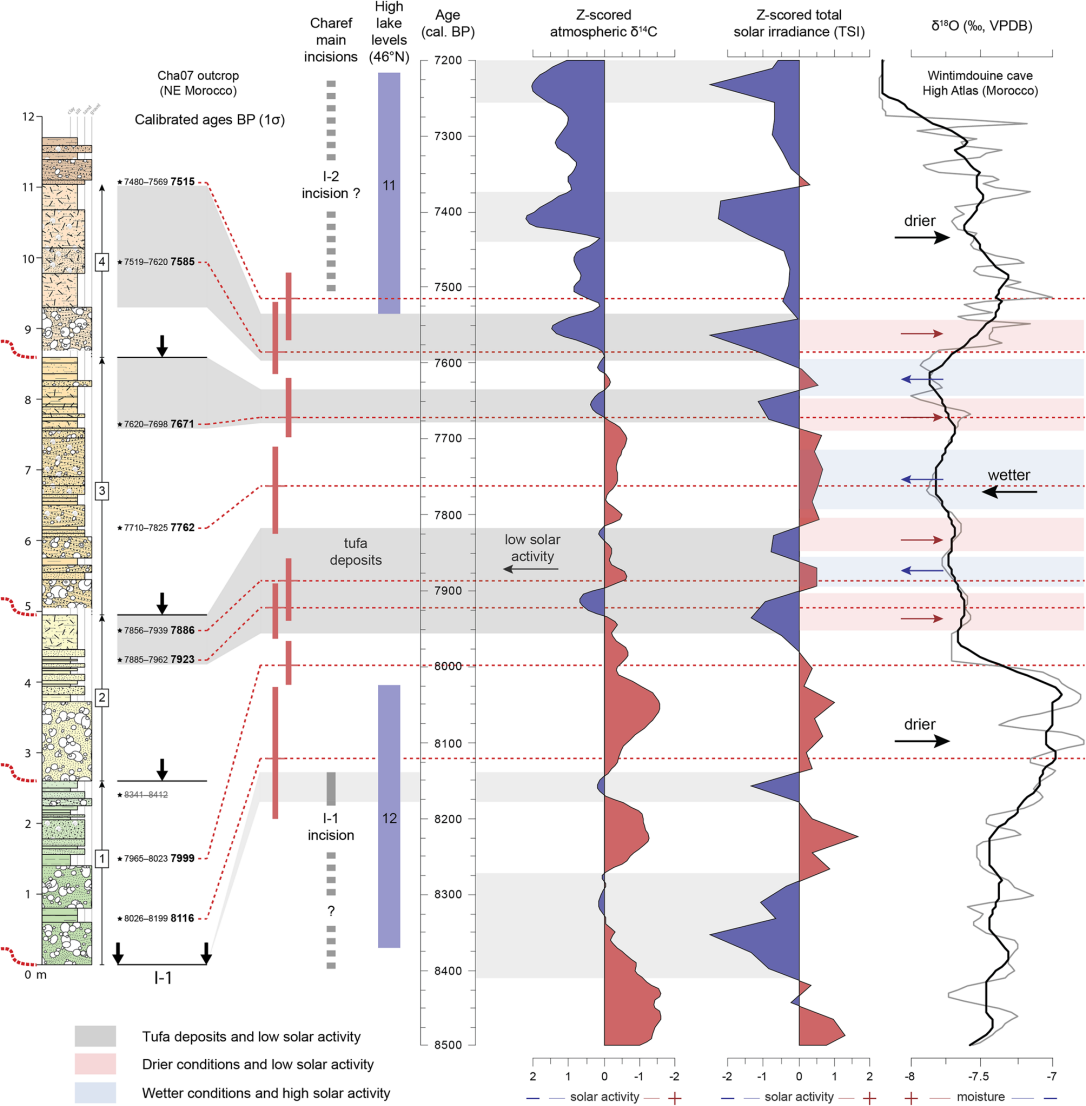
Comparison of the Charef record with mid-European high lake levels^38^, the speleothem record from the Wintimdouine cave in the High Atlas^14^ (δ^18^O values in grey, and moving average over 13 points following a 5-year resampling in black using Analyseries software), and the main solar proxies: Z-scored δ^14^C values^52^ and total solar insolation (TSI, W m^−2^) derived from ^14^C and ^10^Be radionuclides^37^.

The timing of hydrosedimentary changes such as those at the Early–Mid-Holocene transition still need to be discussed. The first major incision (I-1) is marked by a downcutting of at least 4 m and widespread erosion of the top of F2 (Fig. 6). This incision is responsible for a sedimentary hiatus from 8900 to 8150 cal. BP, probably due to the transition from the drier conditions that prevailed during the 8.2 ka RCC in the SW Mediterranean to the return to wetter conditions^13,14^ (Fig. 7). Such causality was already suggested for the Sahelian belt, where, at the same time, a major incision of the Yamé valley associated with a sedimentary hiatus occurred between 8760–7800 cal. BP^31^. Then, four periods of aggradation marked lasting fluvial instability up to 7500 cal. BP. This period ends with a second major incision (I-2), assumed by the Cha07 archive and recognised in two other records, which occurred between 7500 and 7400 cal. BP and which cut into the Cha07 infilling. Its regional extent confirms the role of external climate forcing on the processes of avulsion and paleochannel formation. This event is probably related to the 7.6–7.3 ka RCC. Indeed, this RCC, which seems to have followed a similar hydrological pattern to the 8.2 ka event at a Mediterranean scale, with drier anomalies recorded south to 40°N^36^, could be linked to the last of the proglacial lake outbursts into the Atlantic and the final melting of the LIS^5^, and a grand solar minimum^37^. Both major incisions correspond to ice-rafted debris (IRD) peaks (5a and 5b^3^), mid-European high lake levels^38^, and δ^18^O depletion in the speleothem record from the Carburangeli cave in Sicily^13^ (Fig. 7). In general, the Cha07 record shows very good correlations with the high-resolution palaeoprecipitation record in the High Atlas^14^: the filling of the palaeochannel occurred during a wet period, preceded and followed by the two incisions I-1 and I-2 corresponding to the arid conditions of 8.2 and 7.6–7.3 ka RCCs (Fig. 8).

During this interval, palaeoecological analyses document rather stable conditions during the fluvial sequence S-1, with the presence of tall grasses and riparian forest (8150–7975 cal. BP, Fig. 4). Less stable and drier conditions are noted during S-2 (7925–7850 cal. BP). Lasting hydrological activity (S-3) marked by dynamic fluvial conditions and habitat instability in the malacological record is evidenced between 7800 and 7700 cal. BP (Fig. 4, Supplementary Fig. S10), while pollen data marks the return of riparian forest (Supplementary Fig. S11). These features may result from increased water availability and durability in the channel, which is coherent with the centennial wetter climatic conditions recorded in the Alboran Sea MD95-2043 site ca. 7900–7700 cal. BP^11^, the southern Adriatic Sea MD90-917 site at 7800–7700 cal. BP^10^, the contrasting drier conditions recorded in the Alpine lake levels^38^, and a higher fire regime in the pre-Alpine catchments at 7800–7700 cal. BP^18^. The development of a thick tufa deposit filling the channel ca. 7600–7500 cal. BP implies continuous but reduced flow energy (Fig. 4)*.* Tufa precipitation is associated with the development of a persistent population of *Potomida littoralis,* which prefer calcareous waters, and whose well-preserved shells indicate low energy stream conditions (Supplementary Fig. S10). At the same time, the higher diversity and slight increase in xerothermic molluscs indicate sclerophyllous vegetation on adjacent river banks.

Interestingly, several western Mediterranean records evidence a climatic shift ca. 7500 cal. BP, which is consistent with the second I-2 incision in Moulouya at that time (Figs. 5 and 6). While the centennial-scale 7.6–7.3 ka RCC induced drier conditions in the southern Mediterranean and Africa^11,14,36^, on the millennial scale, it is followed by a long-term climatic amelioration. Indeed, wetter conditions are evidenced by thermo-Mediterranean low fire activity south of 40°N from 7500 to 5000 cal. BP^39^ and a mesophytic optimum in SE Iberia ca. 7500–5200 cal. BP^12^ (Fig. 7). This vegetation response is identified in the Alboran Sea MD95-2043 record, where, after a decline due to the 7.6–7.3 ka RCC, temperate and Mediterranean forest remains high up to 5400 cal. BP^11^. The nearby 293G site records a lasting drop in riverine supply from 7400 to 4500 cal^40^. Similarly, several phases of floodplain stability are evidenced in Iberian rivers, with regional soil formation in the course of the 7th and 6th millennia^21^. These indices demonstrate clear afforestation and increasing floodplain stability associated with the return of humid conditions ca. 7500 cal. BP and their persistence until ca. 5000 cal. BP in the southern Mediterranean, processes coherent with the orbital-scale AHP conditions prevailing in the Moulouya basin^25^. These indices also reflect the sedimentary hiatus in the floodplain deposits of the upstream Moulouya archives from 7500 to 6500 cal. BP, and from 7500 to ca. 5000 cal. BP in channels within the river bed (Fig. 7).

In this respect, we assume predominant effects of an insolation-driven wetter climate and subsequent vegetation response coupled with an initial incision during the drier 7.6–7.3 ka RCC. Indeed, the development of vegetation cover would have stabilised hillslopes and river banks, thereby preventing erosion and runoff. Alluviation would be reduced because of the weaker sediment yield, and possibly constrained within the stream bed because of the entrenchment of the river, before being washed out by floods. Unfortunately, palynological studies for this period are not available for the High Plateaus region. Comparisons with the Middle-Atlas records should be performed with caution, because elevation becomes the principal factor. Nevertheless, expansion of a conifer/sclerophyllous mixed forest ca. 7000 cal. BP is recorded at the Tigalmamine and Ait Ichou lakes (~ 1620 and 1560 m a.s.l.), whereas it appears later at ca. 6300 cal. BP at the Sidi Ali lake (~ 2080 m a.s.l.)^41,42^.

Moreover, it has been noted that particularly strong incision occurred during the transition from dry periods to more humid ones because of a weakening of landscape stability followed by increasing precipitation^31^. This pattern is particularly relevant in the Moulouya basin, where stronger incision during the 7.6–7.3 ka RCC occurred under centennial drier conditions followed by a return to wet AHP conditions. This view is supported by the identification of a flooding deposit ca. 7400–7300 cal. BP at the bottom of the Djamila outcrop in Lower Moulouya^43^, which is followed by soil formation. These particular conditions could also explain the widespread flooding period ca. 7500–7100 cal. BP across the Mediterranean^16^.

#### Holocene deglaciation, insolation, and solar forcing

We see that this prolonged fluvial instability at the Early–Mid Holocene transition mirrors a complex major hydrological change in the northern hemisphere at ca. 8000–7000 cal. BP^6^. Three main forcing mechanisms have been suggested: orbital-driven insolation, end of deglaciation, and solar activity. The final LIS disappearance was marked by the last proglacial lake outbursts into the North Atlantic during this interval, and the subsequent rise of the RSL at 7600 cal. BP^5^, probably inducing the Mediterranean RSL slowdown from 7500 cal. BP onwards^44^. Thus, the melting of the LIS altered the AMOC and NAO-type atmospheric circulation, favouring a reorganisation of the Icelandic Low and Azores high pressure cells, which led to a northward movement of westerlies during the Early–Mid Holocene transition^7^. This shift is supported by the northward migration of the Azores front^45^, reduced coastal upwelling off western Africa^46^, and intensification of the subpolar gyre circulation^47^ that started ca. 8500–8000 cal. BP (Fig. 7). The Charef fluvial instability and major hydrological change also coincide with a lasting decrease in solar activity, called the grand solar minimum, which is indicated by ^14^C and ^10^Be radionuclides from tree-ring and ice-core records^37^.

Throughout the Holocene, the effect of solar forcing on climate variability is evident. Clear correlations between solar activity, monsoon strength, North Atlantic IRD events, and the El Niño–Southern Oscillation^3,48^ have been identified for the Early Holocene. Studies in north-central Europe indicate a major solar impact on continental hydroclimatic conditions during deglaciation, with concurrent reduced solar activity and meltwater pulses^49^. In this manner, during the 8.2 and 7.6–7.3 ka RCCs, the last outbursts and simultaneous decreases in solar activity were concurrent with wetter conditions to the north and drier conditions to the south of the western and central Mediterranean due to the enhanced strength of the Atlantic westerlies^6,27^. With the end of deglaciation, climate variability appears more complex after 7000 cal. BP, because of regulation by oceanic and atmospheric circulation patterns^11^. During the Mid and Late-Holocene, solar forcing and NAO-like atmospheric variability could be the prominent factors driving centennial-scale climatic events in the Mediterranean area^6^. Indeed, wetter conditions evidenced by enhanced flooding and lake-level highstands at mid-latitudes, and associated with IRD events and low solar activity, are in accord with NAO-type contrasting hydrological patterns^3,27^. This assumption is supported by high-resolution records that demonstrate direct solar forcing on hydrological conditions in central Europe, i.e., a southward shift of the North Atlantic storm tracks that resulted in increased rainfall during the Homeric solar minimum (ca. 2700 cal. BP)^50^. This solar-atmosphere linkage is thought to be induced by the so-called ‘top-down’ feedback mechanism proposed by modelling studies^51^, which suggest that important changes in UV radiation disturbed the stratospheric polar vortex and tropospheric jet streams, which finally affected the Hadley Cell circulation and strength and location of North Atlantic storm tracks.

The high-resolution Cha07 record provides detailed information on hydrological responses of the river catchment to climatic variations between the 8.2 and 7.6–7.3 ka RCCs. Our fluvial record suggests a probable forcing of short-term solar variability on the Moulouya river activity during this interval. Indeed, the centennial to multi-decadal variations in paleoprecipitation recorded in the High Atlas^14^ are very well correlated with fluctuations in solar activity (TSI)^37,52^ (Fig. 8), which implies the role of solar forcing on the rainfall regime in the region. These variations follow the De Vries-Suess (~ 210 yr) and Gleissberg (~ 90 yr) cycles, which are both tied to solar variability^51^, and are recognised as modulators of flood frequency in central Europe^53,54^. In Morocco, high-resolution records from the Middle Atlas also suggest solar forcing of hydrological changes through the identification of the De Vries-Suess cycle^55^. We show that the Cha07 tufa deposits are contemporaneous with phases of low TSI and slight decreases in precipitation (Fig. 8). Within the highly dynamic channel environment, we suggest that these tufa deposits should be interpreted as phases of stability that reflect reductions in solid and liquid discharges, linked to multi-decadal decreases in rainfall in the High Atlas and solar activity, knowing that the general humid climatic conditions between the 8.2 and 7.6–7.3 ka RCCs are met for the construction of tufa deposits. We hypothesise that, at the Early–Mid Holocene transition, the hydrosedimentary dynamics of the Charef River were paced by solar variability through a top-down mechanism. The short-term shrinking of the Hadley Cell and southward location of the North Atlantic storm tracks could have also favoured cyclogenesis over NW Africa, leading to more-intense convective storms on the High Atlas and the subsequent rapid hydrogeomorphologic changes recorded in the Charef River. This view could be related to the Mar Menor record in SE Iberia, which demonstrates Mid–Late Holocene storm events coincident with cold periods, and suggests that cyclogenesis variability was related to both AMOC and solar forcing^56^.

Ultimately, our record supports the end of deglaciation coupled with orbital-driven decreases in summer insolation and solar activity variations as the main forcings to explain the millennial hydrological instability in the western Mediterranean. Such relationships raise questions about the role of grand solar minima on the hydroclimatic variability since the Early–Mid Holocene transition. Moreover, we point out the necessity of investigating river catchments at different spatial and temporal scales, even at very high temporal resolution, to perceive millennial to multi-decadal hydrological changes.

## Conclusion

After multi-millennial stability during the Early Holocene, the Cha07 palaeochannel record demonstrates rapid geomorphological evolution of the Charef floodplain ca. 8200–7500 cal. BP, marked by high channel activity and major river incisions. This long-term fluvial instability sheds light on the concurrent sedimentary hiatus in NE Moroccan alluvial deposits and the lack of preserved records in North Africa, addressing these long-unresolved issues. The roles of two Rapid Climatic Changes (RCCs), i.e., the 8.2 ka and 7.6–7.3 ka events, are highlighted. We confirm that RCC-induced drier climatic conditions, followed by wet conditions, led to enhanced fluvial activity in the Moulouya basin, and probably also in other arid environments in North Africa.

Furthermore, we document that the global-scale 8.2 ka event, which was surprisingly missing from southern Mediterranean fluvial records, had a prominent impact on the Moulouya river system. We suppose that the absence of river responses to this RCC in other fluvial syntheses stems from the major disruption of the 8.2 ka event on North African river systems. The widespread flooding period ca. 7500–7100 cal. BP across the Mediterranean is evident through a second major incision in the Moulouya basin, and the subsequent sedimentary hiatus at a regional scale may reflect the return to a Mid-Holocene hydrological optimum, thus emphasising the importance of taphonomic consequences of RCCs in fluvial contexts and associated archaeological archives.

The end of deglaciation marked by proglacial lake outbursts, and concurrent decreases in summer insolation and solar activity, appear as the main drivers of these hydroclimatic changes. Indeed, the final Laurentide ice sheet melting in the course of the 8th millennium led to a reorganisation of oceanic and atmospheric circulation patterns, affecting western Mediterranean hydrology during the Early–Mid Holocene transition.

Moreover, the hydrosedimentary changes of the Charef River between 8200 and 7500 cal. BP seems to have been particularly sensitive to centennial variability of solar activity (Suess-deVries and Gleissberg cycles), through fluctuations in regional precipitation, and highlights the potential role of solar forcing on fluvial morphogenesis in North Africa.

## Methods

### Chronology

Nine AMS radiocarbon datings were performed on charcoal materials. All these radiocarbon-derived ages were calibrated using IntCal20^52^. The Cha07-log3b-45-1 date (8205–8445 cal. BP) from the top of S-1, which was older than all other dates (Supplementary Table S1), was excluded because the charcoal was located in a coarse sandy unit and could correspond to older charcoal carried by a flood. An age model was performed using Oxcal v.4.4^57^.

### Loss-on-ignition

The loss-on-ignition (LOI) method was used to determine the organic matter content (OM, % of the bulk mass) of 117 samples. Samples were dried for 12–24 h at 106 °C. The OM content was combusted at 550 °C over 5 h^58^.

### Malacology

A total of 53 malacological samples with a constant volume of 4 L were acquired by adapting the sampling interval to the lithostratigraphy (Supplementary Fig. S1). Coarse and gravelly levels were not sampled for mollusc analysis. Each volume of sediment was wet sieved with a 500-μm mesh. The residues were sorted microscopically to extract and identify shells. Complete shells and identifiable fragments were counted to reach a minimum number of individuals for each sample. Recent literature and targeted papers on the Moroccan fauna were consulted to identify the most common Mediterranean land and freshwater Mollusca, as well as local species^59–61^. Because of the very high abundance of shells, only odd-numbered samples were analysed in this study. The mollusc assemblage ecological analysis was based on an adaptation of the Puisségur^62^ classification and habitat descriptions available in the literature.

### Palynology

The sediment of 47 samples was sieved using a 400-µm sieve to remove the coarsest fraction. A chemical treatment (HCl acid, 10% NaOH, and acetolysis) was used to extract the sporo-pollinic material, followed by flotation in heavy liquid (d = 2) and 160 + 10-µm sievings. A Leitz Biomed photonic microscope (500 × magnification) was used to identify pollen, spores, and non-pollen palynomorphs (NPP). Identifications relied on the pollen reference collection of IMBE (CNRS, Aix-en-Provence, France), pollen photographic atlases^63^, and articles on NPP^64^. The pollen percentages were calculated on a pollen sum (PS) including all plants except Cryptogams and NPP. The PS varied from 179 to 388. Of the 47 samples analysed, only 8 were polliniferous, all of which were located in the lower part of the series. The pollen concentration (weighting method)^65^ was low, and varied from 47 pollen/g (sample 565) to 9 pollen/g (sample 402).

## Supplementary Information

Supplementary Information.
