# Supplementary material for: First fluvial archive of the 8.2 and 7.6–7.3 ka events in North Africa (Charef River, High Plateaus, NE Morocco)
Source: Sci Rep. 2022 May 11;12:7710. doi: 10.1038/s41598-022-11353-y (PMC9095645; doi:10.1038/s41598-022-11353-y)
Supplement: Supplementary file 1 — Supplementary Information. [file 41598_2022_11353_MOESM1_ESM.docx]

Supplementary material for

**First fluvial archive of the 8.2 and 7.6–7.3 ka events in North Africa (Charef River, High Plateaus, NE Morocco)**

Bruno Depreux^1,2^*, Jean-François Berger^3^, David Lefèvre^1,2^, Quentin Wackenheim^4,5^, Valérie Andrieu-Ponel^6^, Sylvia Vinai^2^, Jean-Philippe Degeai^1,2^, Abderrahmane El Harradji^7^, Larbi Boudad^8^, Séverine Sanz-Laliberté^1,2^, Kristell Michel^9^, Nicole Limondin-Lozouet^3^

1 Univ. Paul Valéry Montpellier 3, CNRS, UMR 5140 Archéologie des sociétés méditerranéennes, Campus Saint Charles, F-34000, Montpellier, France

2 LabEx Archimède, Univ. Paul Valéry Montpellier 3, Campus Saint Charles, F-34000, Montpellier, France

3 Univ. Lyon, Université Lumière Lyon 2, CNRS, UMR 5600 Environnement Ville Société, F-69635, Lyon, France

4 Univ. Paris 1, UPEC, CNRS, UMR 8591 Laboratoire de Géographie Physique, F-92195, Meudon, France

5 Univ. Paris 1, CNRS, UMR 8215 Trajectoires, F-75004, Paris, France

6 Institut Méditerranéen de Biodiversité et d’Ecologie Marine et Continentale, Aix Marseille Univ., CNRS, IRD, Technopôle de l’Environnement Arbois-Méditerranée, F-13545, Aix-en-Provence, France

7 Univ. Mohamed 1^er^, Oujda, Morocco

8 Univ. Mohammed V, Rabat, Morocco

9 Univ. Lyon, ENS de Lyon, CNRS, UMR 5600 Environnement Ville Société, F-69342, Lyon, France

*corresponding author (bruno.depreux@univ-montp3.fr)

**The supplementary material file includes:**

- Supplementary information
- Supplementary figures S1–S11
- Supplementary tables S1–S3
- Supplementary references

**Supplementary information**

**Mapping and characterisation of the sedimentary formations**

Only the Cha07 outcrop is presented here, as the paper focuses on the response of the hydrosystem to the Early and Middle Holocene transition, but it is part of a wider study of the Holocene sedimentary formations in this area, in the Aïn Beni Mathar (ABM) basin, and even more widely in the upstream basins of the Moulouya (Fig. S1). Indeed, numerous surveys have been carried out in the rivers of the ABM basin and in the Ksabi basin in the Middle Moulouya, within the framework of research programmes (Paléomex/Mistral of the CNRS / Paléomar of the LabEx ARCHIMEDE). This has enabled the development of a multiscalar approach to the fluvial archives of NE Morocco, by comparing two similar areas (ABM and Ksabi basins) more than 300 km apart, and to study them in detail using a systemic approach (several rivers within which several sections for each formation). Thus, the ABM basin has been extensively prospected and studied, and we present in Fig. 1 and Fig. S2 only the sector concerned by the issues of this paper. The other formations in the Charef study area have therefore been studied and are presented briefly here.

The construction of the geomorphological map is based on:

- The topographic data acquired by dGPS surveys in the field, and by the creation of a DTM from a photogrammetric model (produced with the Metashape software) made from our own aerial drone image acquisitions ;
- The surface observations of the different sedimentary formations, as shown on map Fig. S2 illustrating the location of these observation points.

Two stratigraphic cross-sections of the valley (Figs. S3 and S4) and a summary table (Fig. S5) show the organisation of these different sedimentary formations and their main architectural and lithological characteristics.

Fine wetland deposits (F2) accumulated in the southeast part of the river section, and are developing eastwards, whereas there is an underlying carbonated massive sands (F1) outcrop in the northwest part (Fig. S2). F2 corresponds to the climate-driven extensive Early Holocene formation recognised in the upper and middle reaches of the Moulouya catchment, which reflects the orbitally-induced African Humid Period (Depreux et al., 2021).

The palaeochannel (Cha07 outcrop) cuts into the F1, F2, and the Red Mudstone formations. Its filling is dated from 8150 to 7500 cal. BP. The chronology of this archive perfectly fills a sedimentary hiatus generalised to the whole of the alluvial archives of NE Morocco, which extends from 8700 to 7300 cal. BP (Supplementary Fig. S6).

A sedimentary gap is subsequently recognised in the study area between 7500 and 5200-5000 cal. BP, which is accompanied by a significant incision and lowering of the base level (Figs. S3 and S4). The first indices of alluviation are recognised at the Cha08 outcrop, located at the base of the terrace of formation F3, 700 m further north than the Cha07 palaeochannel, with channel deposits dated to 5 ka cal. BP (Fig. S7). This new phase of aggradation, from 5200-5000 cal. BP until about 2000 cal. BP, begins at least 1 metre below the present base level with channel deposits. The latter are characterised by sandy layers and gravel layers mixed with rounded clay aggregates. They aggraded after an incision below the current base level into the Red Mudstone formation, and marked the start of a new alluvial formation (F3). This long-term sedimentary hiatus from 7.5 to 5 ka cal. BP mirrors the wetter conditions identified at a regional scale and the possible forcing of afforestation on sediment yield and floodplain stability.

This 9 m high terrace, nested within the bedrock and F1 and F2, is composed of reddish brown silt and sand and poorly developed cumulic palaeosoils. After a further incision a new, undated, cut-and-fill terrace was formed during the last two millennia. Both cross-sections show a very significant current valley incision within the Holocene deposits. Pleistocene fluvial conglomerates sometimes form the alluvial floor and sometimes are already incised. The stratigraphic relationship with the Red Mudstone formation is not clearly established.

**Malacological data**

Malacological data classified by ecological groups are presented in Table S2. The following malacological succession has been analysed on the base of ecological diagrams (Fig. S2) and frequency histograms (Fig. S3). Ecological diagrams are elaborated by considering the proportion of ecological groups by the number of species (Fig. S2 upper part) and, by the number of individuals (Fig. S2 lower part) to better appreciate the palaeoecological analysis (Puisségur 1976).

Malacozone 1 (MZ1, samples M53 to M41)

This malacozone is marked by the consistent occurrence of aquatic taxa rich in species and individuals. Freshwater molluscs represent between 40% and 50% of the species diagram (Fig. S2). The succession shows a high abundance of *Melanopsis* and *Mercuria* taxa, two gastropods that tolerate a wide variety of running freshwater environments (e.g., rivers, irrigation channels, springs). Other aquatic molluscs are broad-ranging species whose habitats include both dynamic and stagnant environments. The terrestrial snails *Oxyloma elegans, Carychium minimum, Vertigo antivertigo,* and *Vallonia enniensis* are identified in Charef, and account for an average of 20% of the specific assemblage of malacozone 1 (Fig. S2). The first three species are common molluscs of marshy areas, whereas the occurrence of *Vallonia enniensis* implies the development of perennial fully-swampy conditions. Xerothermic, open field, and mesophilic taxa reach about 10%–15% of the species assemblage. The xerothermic and mesophilous groups are relatively diverse, whereas the open field molluscs are largely represented by *Vallonia pulchella,* which demands slightly humid conditions. Occurrences of slugs and hygrophilous taxa are occasional, and reflect relatively wet conditions and sparse vegetation. The environment deduced from Malacozone 1 is characterized by predominant aquatic habitats surrounded by a few adjacent palustrine areas and sparse xeric vegetation.

Malacozone 2 (MZ2a, samples M39 to M31 & MZ2b, samples M29 to M19)

Malacozone 2 shows a slight increase in the proportion of aquatic species, which represent more than 50% of the specific assemblage (Fig. S2). *Melanopsis* taxa dominate the abundant and diverse freshwater assemblage. Among the terrestrial snails, palustrine species represent a proportion of around 20%, whereas xerothermic, open field, mesophilous, and slug parts are low (5%–15%). Malacozone 2 is divided into two sub-zones (MZ2a and MZ2b) on the basis of changes that occur in the second part of the zone: i) the occasional occurrence of *Bulinus truncatus* and *Ecrobia* aff *ventrosa ;* ii) the discontinuous presence of *C. minimum* and *V. enniensis*; iii) a slight increase in the proportion of xerothermic molluscs (Fig. S3). Therefore, the mollusc frequencies and composition of malacozone 2b reflect unstable environmental conditions. Malacozone 2 indicates strong fluvial dynamics affecting the sustainability of the terrestrial environments.

Malacozone 3 (MZ3, samples M17 to M1)

Freshwater molluscs still represent 50% of the species proportion and show a higher diversity (Fig. S2); however, the distribution of aquatic taxa differs slightly from the previous zone. Malacozone 3 indicates a well-established population of the bivalve *Potomida littoralis* and the development of two gastropods, *Bulinus truncatus and* *Ecrobia* aff *ventrosa* (Fig. S3). *Potomida littoralis* is a bivalve that prefers calcareous waters; their well-preserved shells indicate low energy stream conditions. The proportion of paludal gastropods is constant, and reaches 20% of the specific assemblage. Xerothermic land snails show a slight increase to around 15%–20%. This group includes *Rumina decollata*, *Sphincterochila, Granopupa granum, Helicopsis* aff. *naini* and *Cochlicella barbara,* which are xero-resistant snails that prefer exposed habitats with sparse vegetation and rocky areas. The mesophilous and open field species proportion is around 10%. *C. tridentatum* occasionally appears at the beginning of the malacozone and is the sole hygrophilous taxon (Fig. S3). Mollusc assemblages in Malacozone 3 reflect the predominance of aquatic environments with small wetlands and xeric vegetation developed adjacent to the riverbanks.

**Palynological data**

Vegetation

The Charef catchment is located in the arid High Plateaus south of the Oujda Mountains in eastern Morocco (Wengler and Vernet, 1992). The dominant vegetation is a steppe with *Alfa tenacissima* and *Artemisia herba-alba*. Towards the south, the steppe is enriched with species adapted to long-lasting drought (*Anabassis aphylla*) and then to desert conditions (*Fredolia aretioides*, *Haloxylon scoparium*). In the north, with an increase in humidity and relief, the vegetation is dominated by *Quercus ilex* forest. On the slopes and piedmont, the forest is sparse and comprises *Tetraclinis articulata*, *Olea* sp., *Pistacia lentiscus,* and relict forests of *Pinus halepensis*. Along the wadis, the vegetation includes woody plants characteristic of arid and semi-arid bioclimates such as *Salix* sp., *Tamarix articulata*, *Nerium oleander*, *Retama sphaerocarpa,* and *Populus alba*.

Description

The pollen data include eight polliniferous samples between 60 and 565 cm depth, at the bottom of the fluvial series of the Charef River. Taphonomic reasons explain the absence of pollen in the forty other samples analysed. The pollen concentration of the polliniferous samples is low (between 9 and 47 pollen/g), but as the taxonomic diversity is high (64 taxa identified in total) for samples from an arid and fluvial depositional environment, it is possible to propose hypotheses on changes in vegetation, climate, and possibly human activities.

According to the evolution of the arboreal pollen rate (AP), that of aquatic plants typical of a more humid climate, and the presence of water in the Charef river, four pollen zones were identified (Fig. S4). LPAZ 1 is characterised by the presence of aquatic (*Myriophyllum verticilatum*) and hygrophilous (*Sparganium/Typha* t.) taxa, cereals, and a range of nitrophilous plants (Cichorioideae, *Plantago coronopus,* and *P. major/minor* t.). The AP rate is low (<30%), with AP values being at their highest in LPAZ 2. This is related to an increase in the rate of deciduous *Quercus* and *Pinus* that could develop within the meso-Mediterranean association, in the Oujda mountains, north of the High Plateaus. The pollen of cereals including rye is noted, as well as high levels of nitrophilous grasses and spores of coprophilous fungi (*Sporormiella*, *Podospora*, Type 200), which are good indicators of the presence of large herds of herbivores. LPAZ3 is characterised by a collapse of AP percentages and a maximum of Chenopodiaceae. There is also a strong decline in *Artemisia* and Poaceae values, while the Chenopodiaceae steppe reaches an optimum in extension. The percentages of *Olea* and *Prunus* sp. are also at their maximum. In LPAZ4, Poaceae and *Artemisia* values rise again, but the most characteristic events in this zone are the new maximum of *Olea*, the extended maximum of nitrophilous herbs, and the new peak of riparian forest (*Tamarix*). High rates of charcoal are also noted.

It is interesting to mention the presence of *Lygeum spartum* (Table S3), which is a thermophilous Poaceae typical of the *Alfa tenacissima* and *Peganum harmala* steppe of North Africa. The pollen of *Picea* was also identified in the lower part of the record (Table S3). *Picea* is a medio-European tree that was present in the recent past (MIS 3 and Holocene) on the French Mediterranean coast (Andrieu, unpublished and Triat-Laval, 1978). Its presence on the southern Mediterranean shore can be envisaged, but new discoveries of this taxon would be necessary to confirm this floristic curiosity. As the pollen was extracted in a hyperbaric clean room and in a Mediterranean environment without *Picea* (Aix-en-Provence region), the hypothesis of pollution from the current pollen rain cannot be envisaged. About thirty years ago, the pollen of *Fagus* was identified in the El Khala series (E. Algeria, Benslama et al., 2010), but the presence of this taxon was less singular than that of *Picea,* insofar as *Fagus* has a complex ecology that seems to accommodate a Mediterranean climate, as indicated by the high values of this taxon in the Holocene pollen assemblages of the northern Mediterranean (Triat-Laval, 1978).

**Supplementary figures**


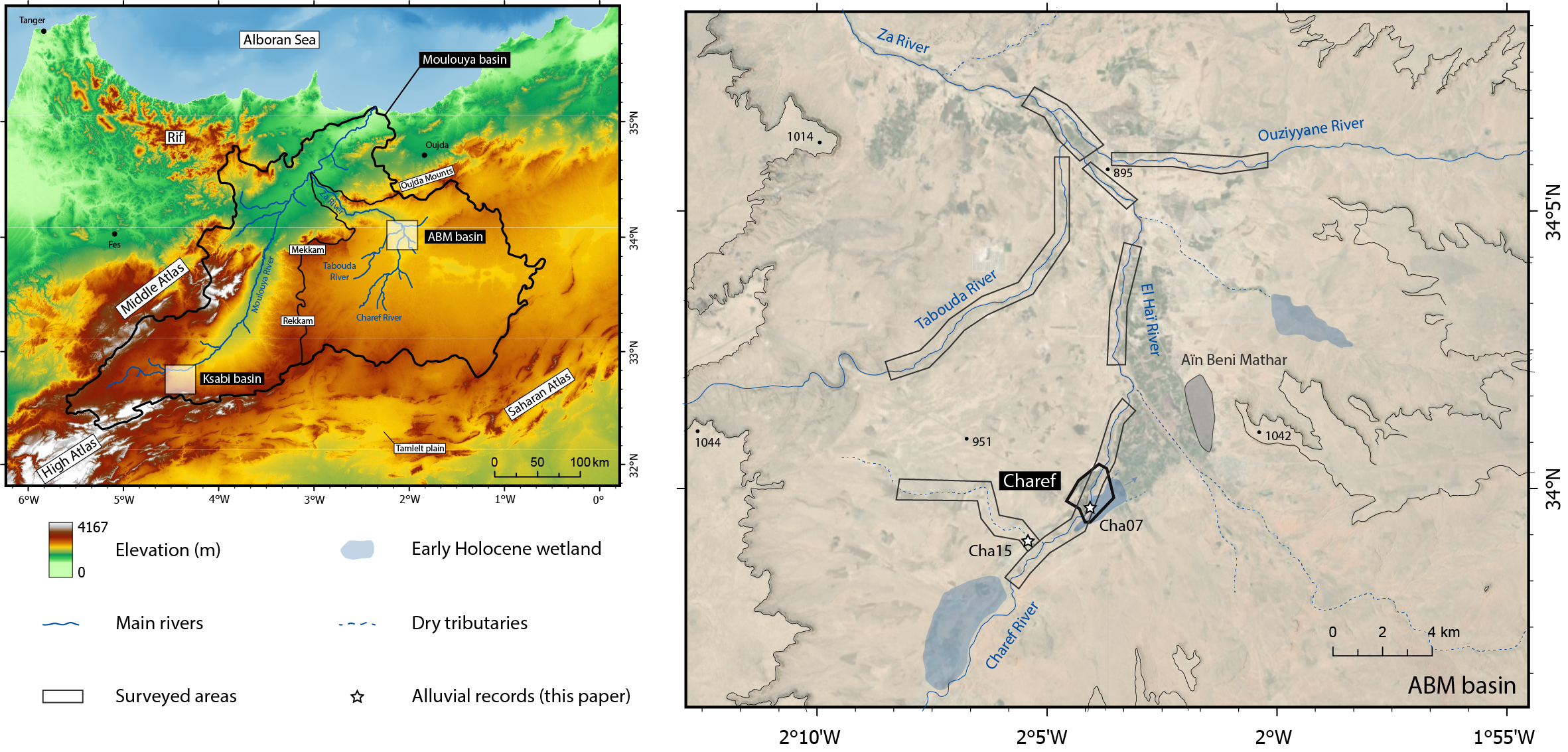


Figure S1. Map of the Ain Beni Mathar basin and location of the Charef study area and of section Cha15 located 2 km upstream in a tributary of the Charef River. Map created by B. Depreux using ArcGIS Pro [2.7] (https://www.esri.com/en-us/arcgis/products/arcgis-pro/).


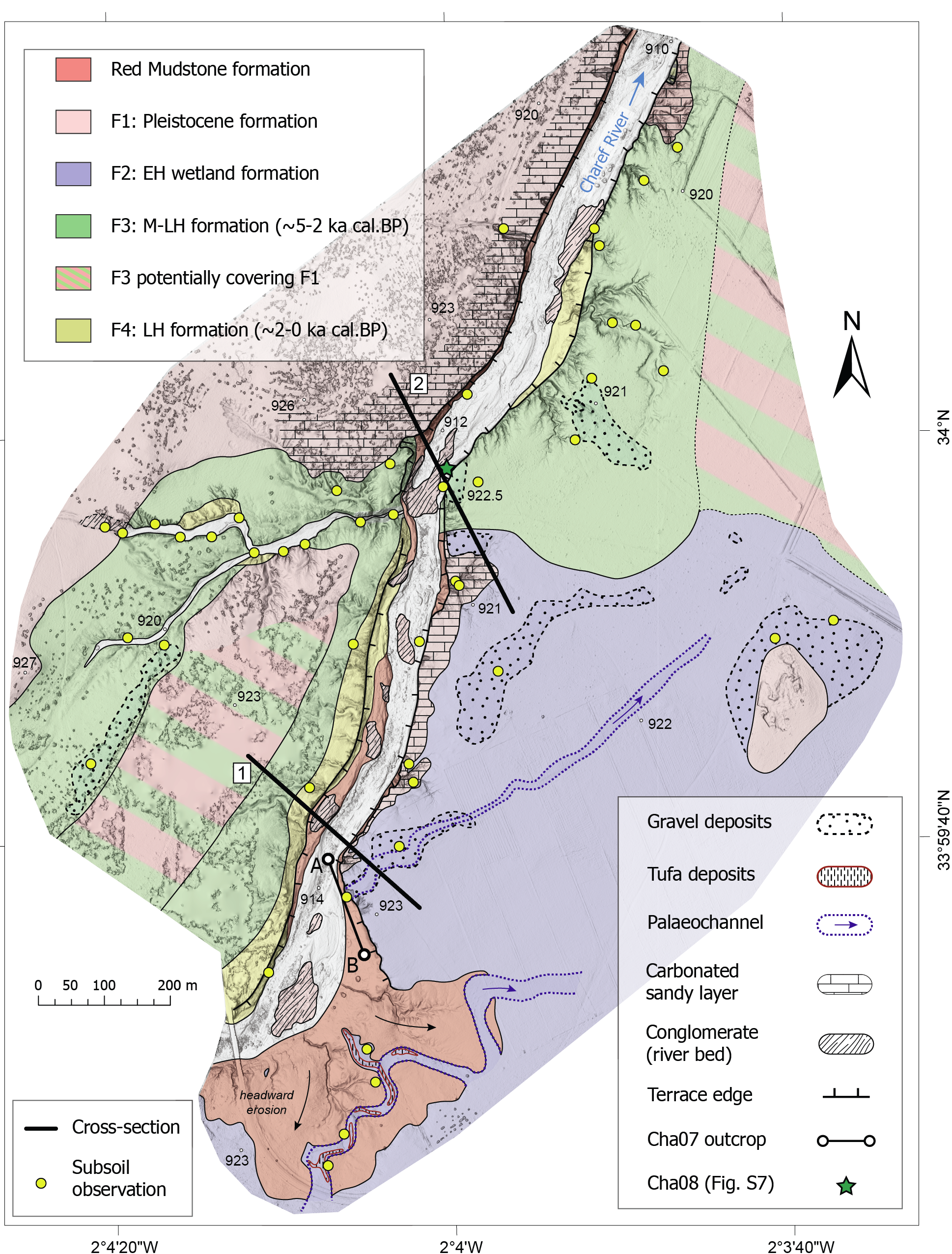


Figure S2. Geomorphological map of the study area with location of the two stratigraphic cross-sections and subsoil observations. Map created by B. Depreux using Adobe Illustrator CC (https://www.adobe.com/).


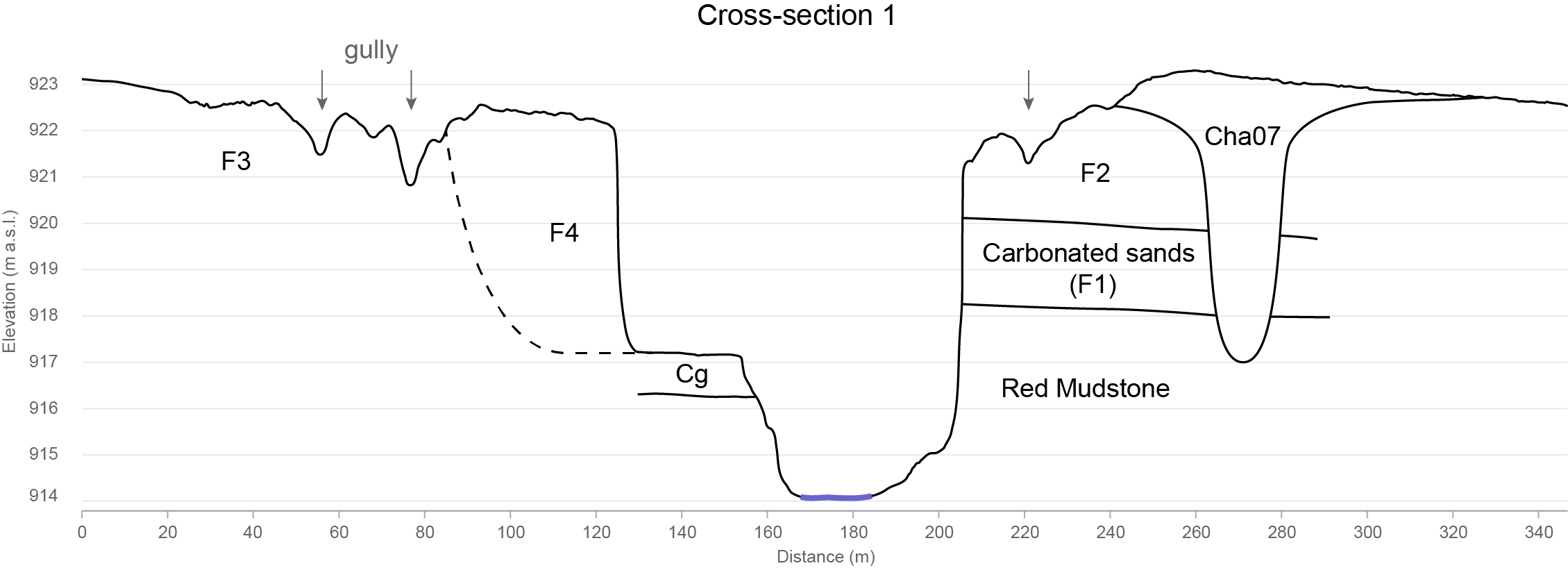


Figure S3. Cross-section N°1 and position of the different sedimentary formations.


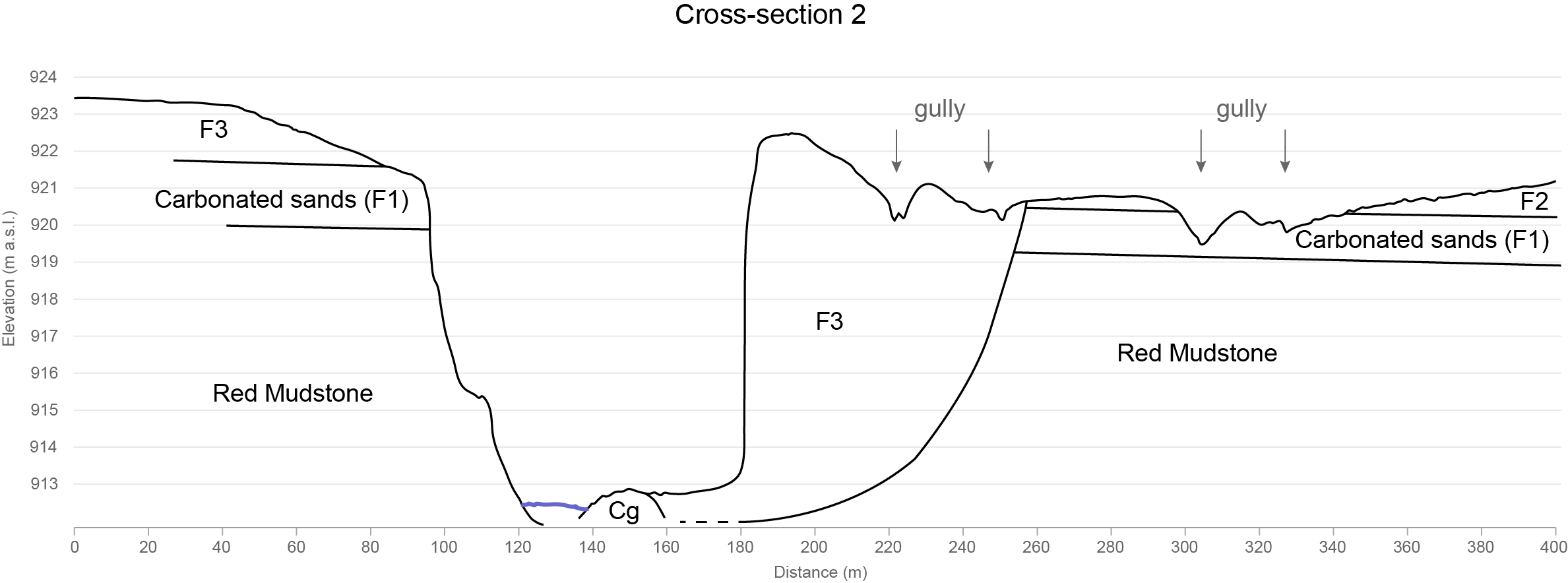


Figure S4. Cross-section N°2 and position of the different sedimentary formations.


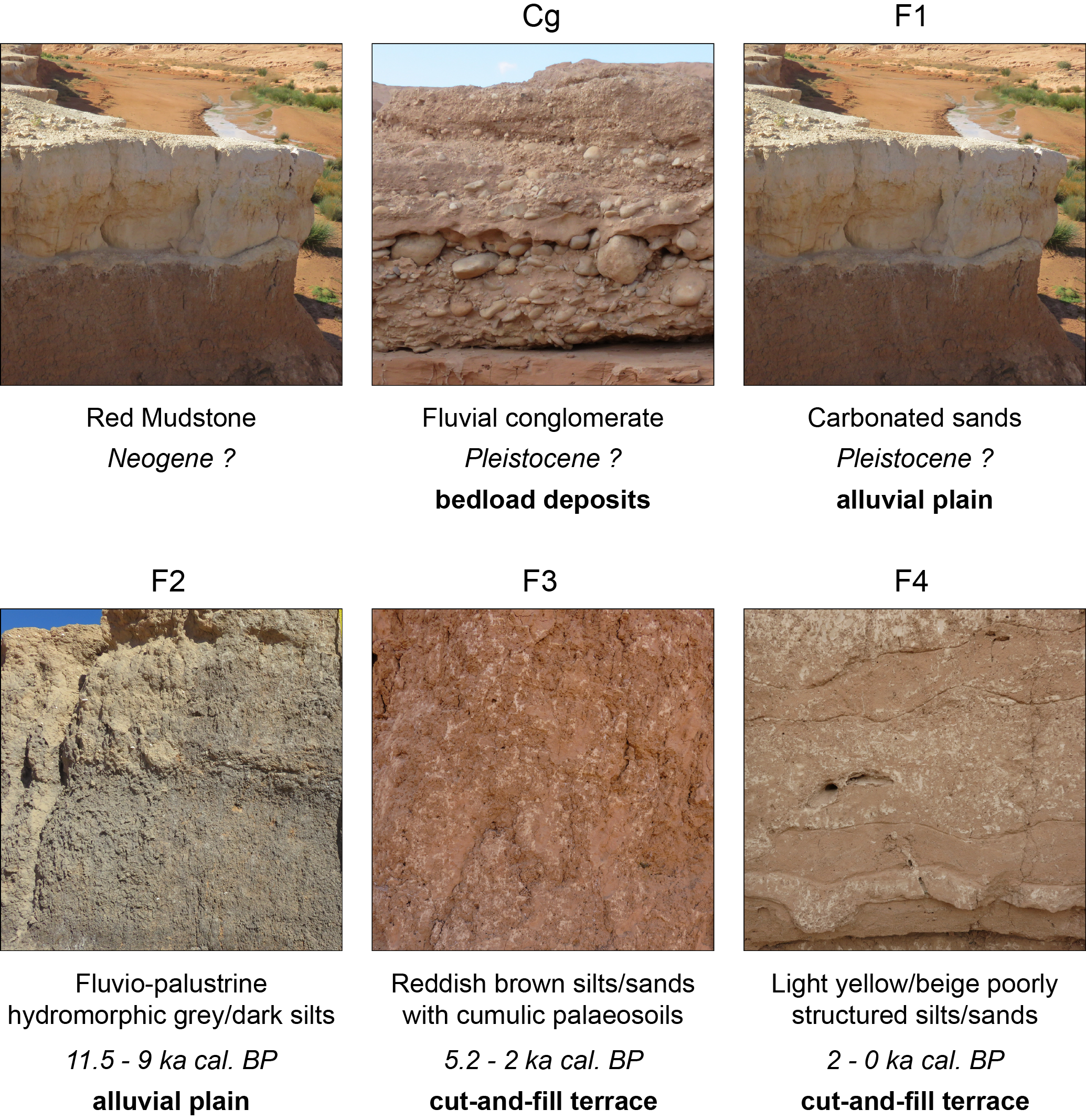


Figure S5. Summary information on the lithostratigraphic characteristics of the different sedimentary formations. Data source: pictures from B. Depreux.


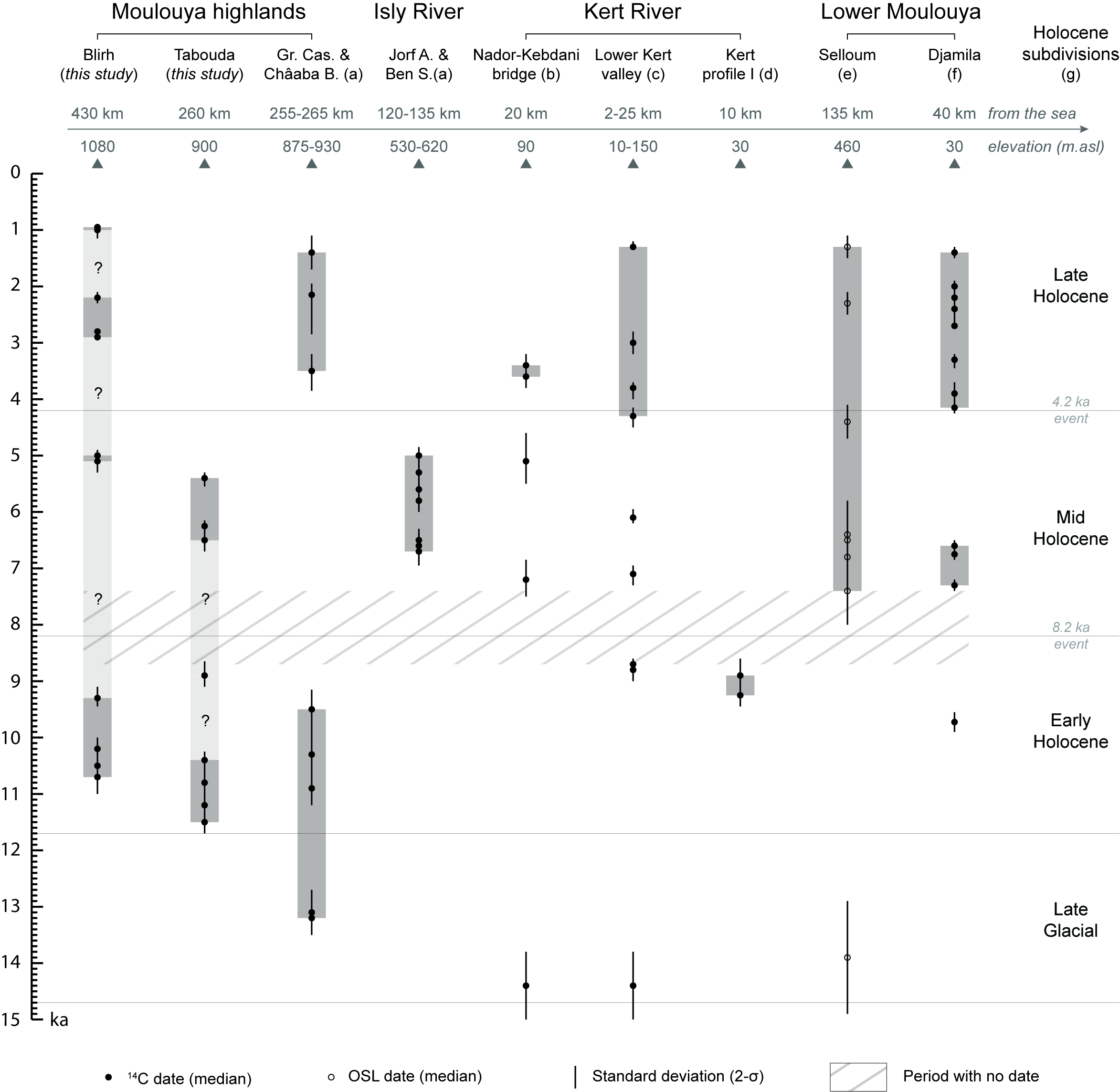


Figure S6. Comparison of alluvial formation chronologies of the upstream studied river sections (Tabouda and Blirh) with other fluvial studies from NE Morocco: the El Hay River and Isly River ((a) Wengler et al., 1994); Kert River ((b) Barathon et al., 2000, (c) El Amrani et al., 2008, (d) Zielhofer et al., 2008); and lower Moulouya River ((e) Bartz et al., 2017, (f) Ibouhouten et al., 2010 and Zielhofer et al., 2010). The hatched band shows that the period from 8700 to 7300 cal. BP, taking into account the error margins, corresponds to a sedimentary gap in all the fluvial archives of NE Morocco. Vertical grey bars indicate continuous sedimentary formation with successive radiocarbon dates. Vertical light grey bars concerning the Tabouda and Blirh outcrops are taken into account possible sedimentary hiatuses. (g) Holocene subdivisions refer to Walker et al. (2018) (after Depreux et al., 2021).


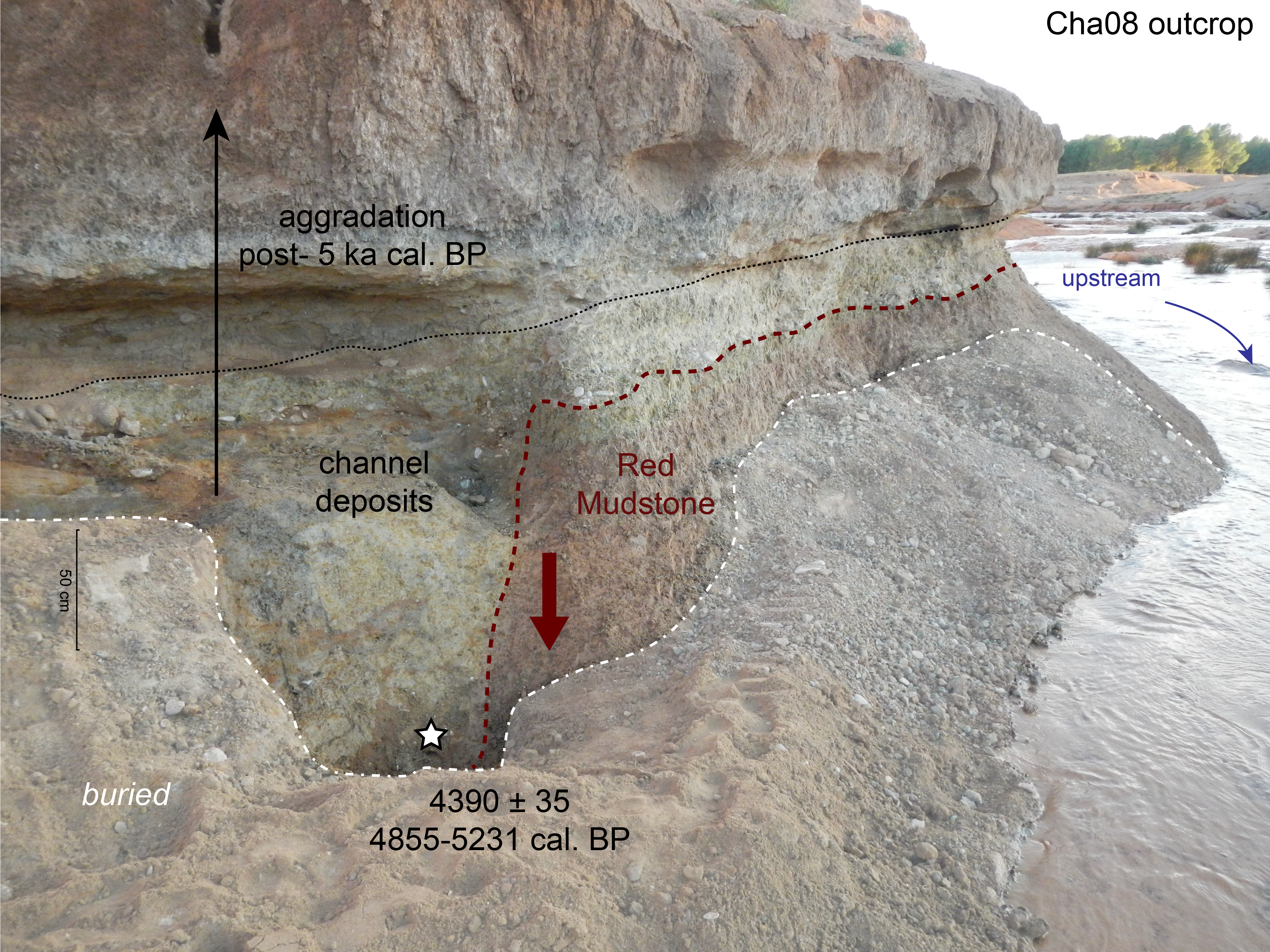


Figure S7. View of the Cha08 outcrop. Data source: picture from B. Depreux.


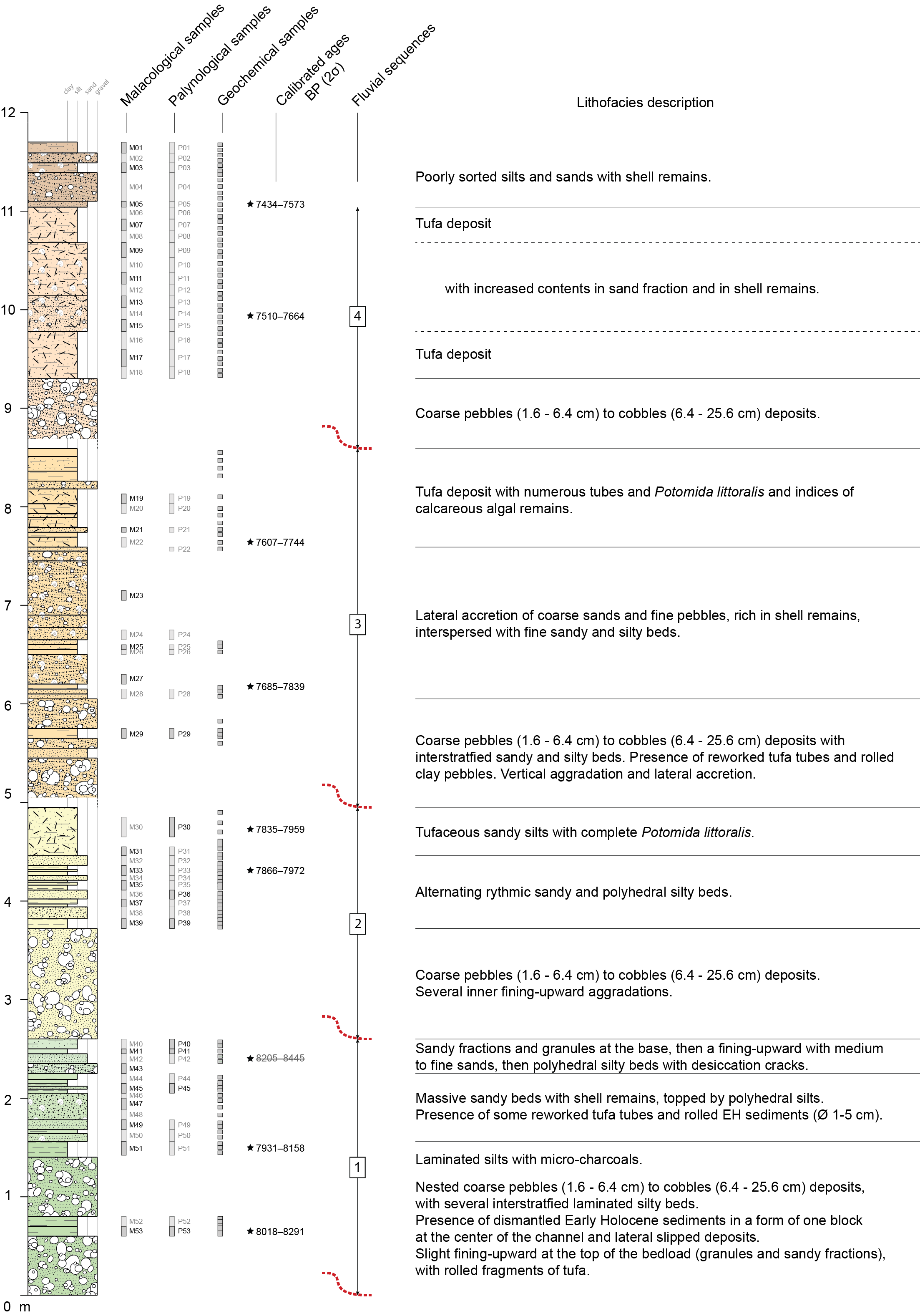


Figure S8. Cumulative stratigraphic log with positions of the malacological, palynological, and geochemical samples (those analysed are in dark grey), and descriptions of the associated lithofacies.


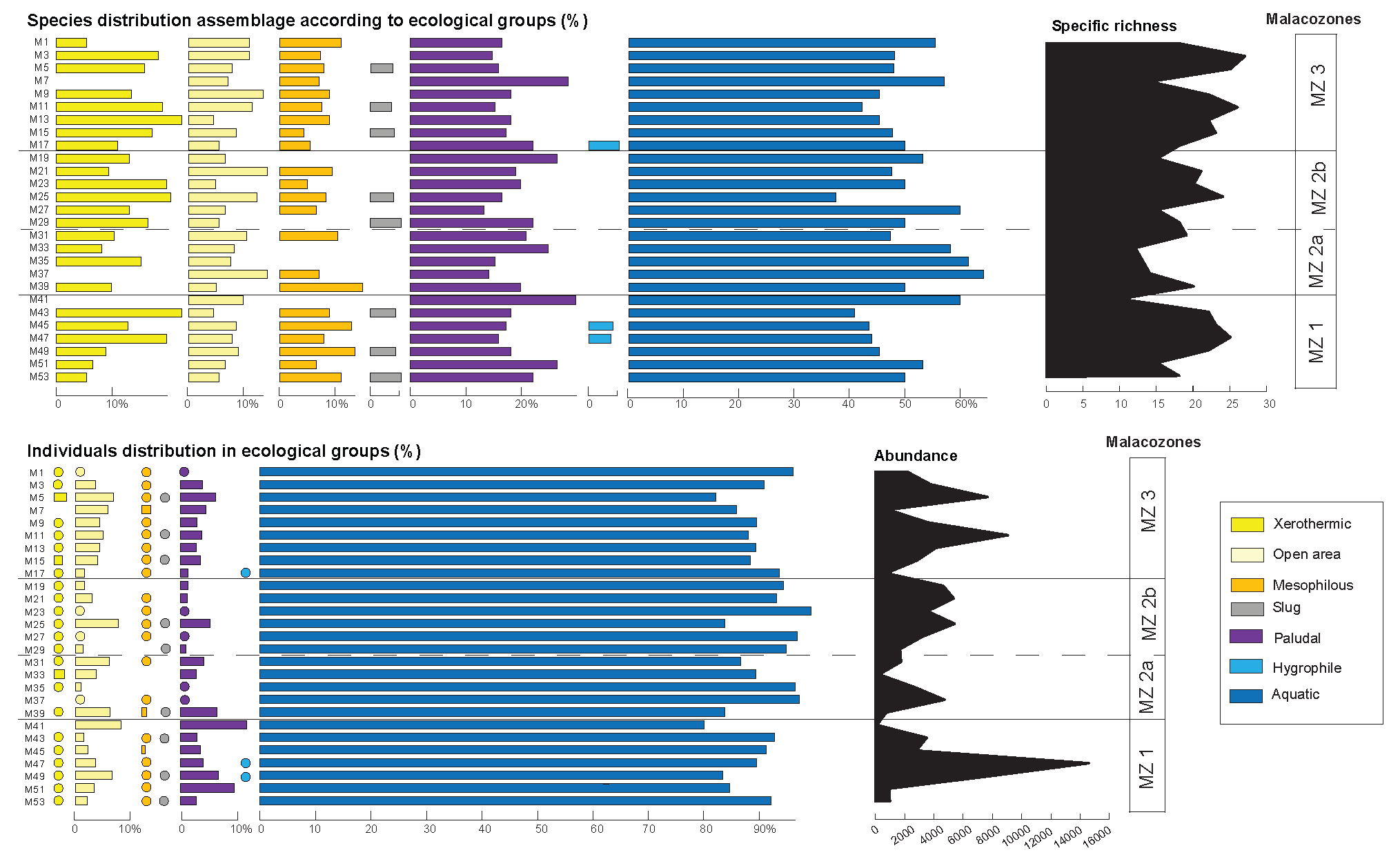


Figure S9. Malacological ecological diagrams of the Cha07 outcrop by species and by individuals.


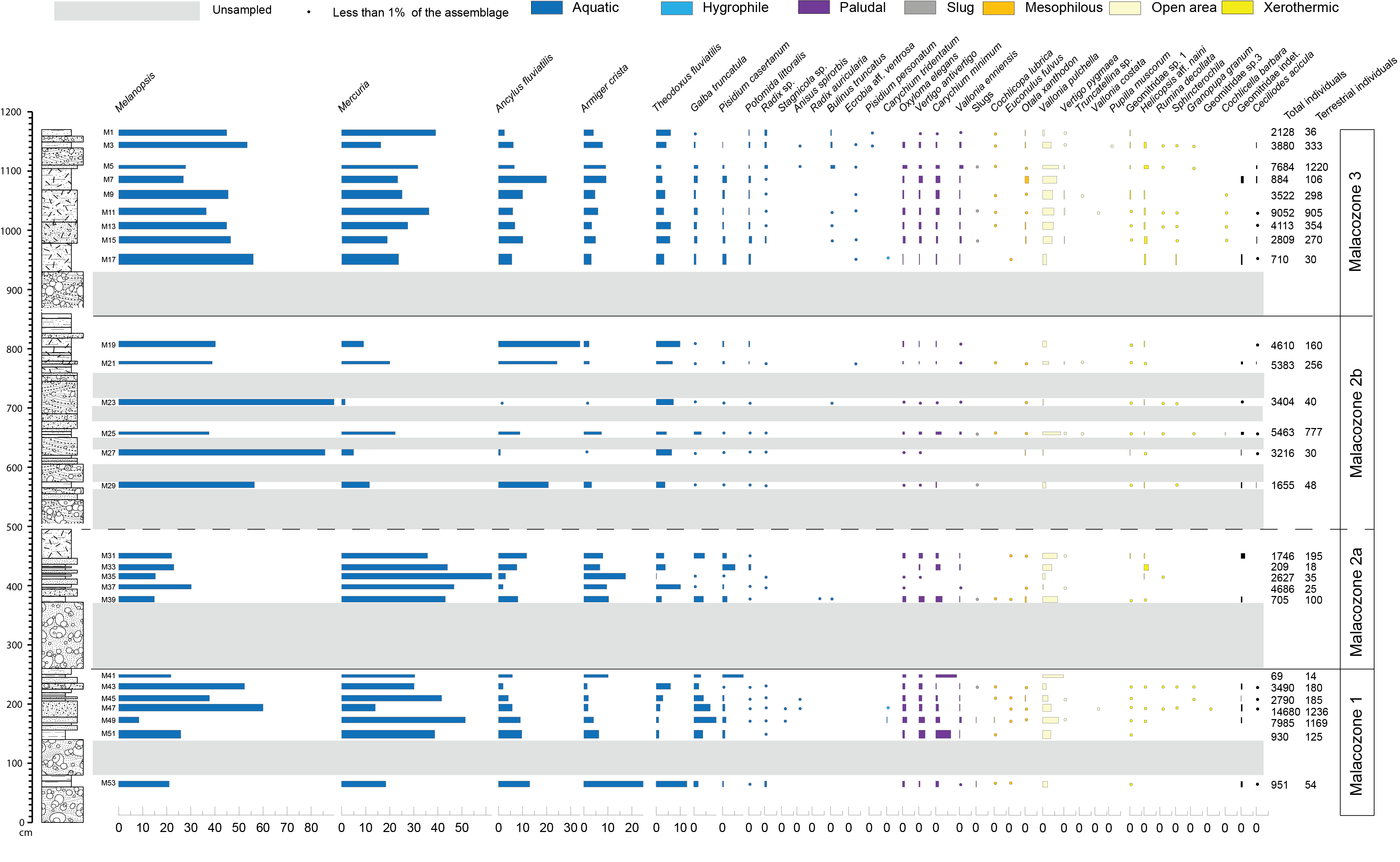


Figure S10. Percentage frequency histogram of the Cha07 outcrop.


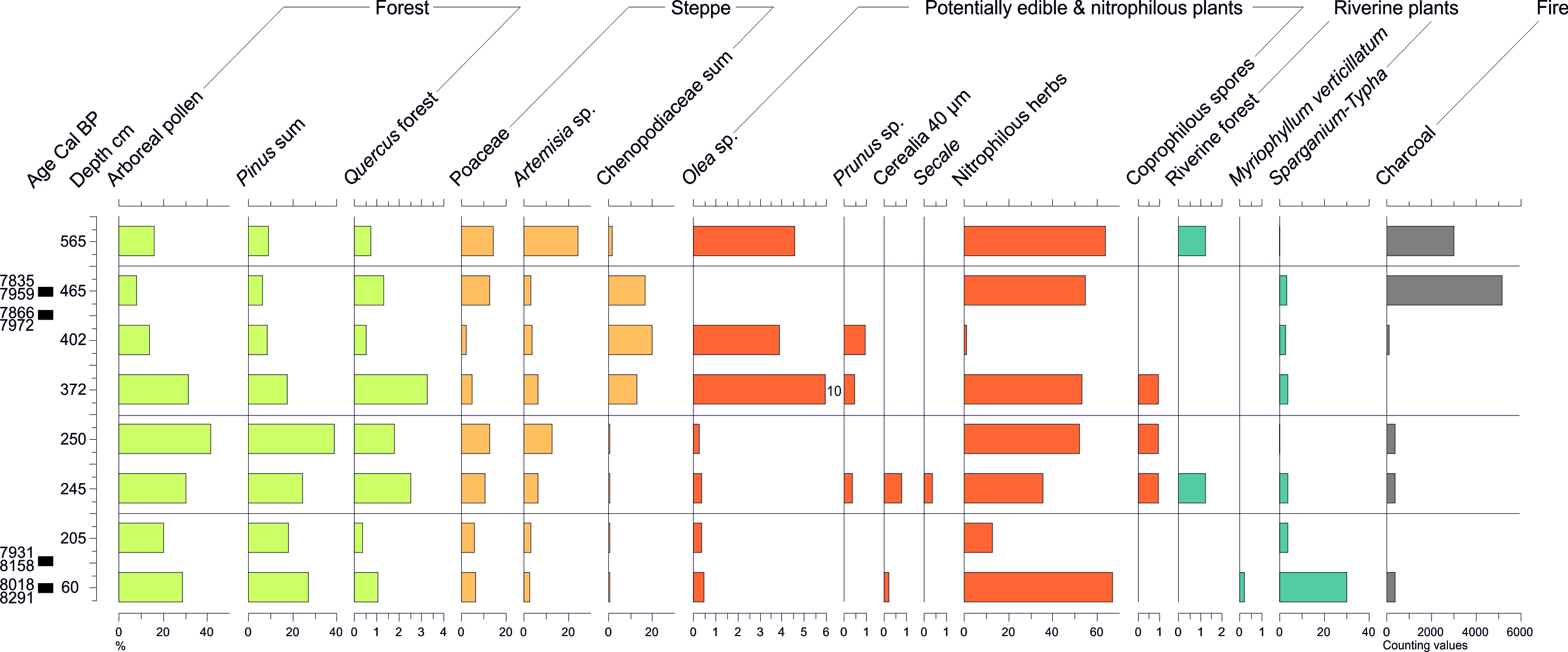


Figure S11. Simplified pollen and NPP diagram of the Cha07 outcrop (equidistant scale).

**Supplementary tables**

| Outcrop | ID code | Lab no. | Depth (cm) | Material | Age 14C BP | Age cal. BP  (2σ) | Modelled age cal. BP (2σ) | Modelled age cal. BP (1σ) | Modelled age cal. BP (median) |
| --- | --- | --- | --- | --- | --- | --- | --- | --- | --- |
| Cha07 | CHA07 log7 182 | Poz-110512 | 1107 | charcoal | 6610 ± 40 | 7429–7569 | 7434–7573 | 7480–7569 | 7515 |
| Cha07 | CHA07 log6 164 | Poz-110514 | 994 | charcoal | 6710 ± 50 | 7485–7668 | 7510–7664 | 7519–7620 | 7585 |
| Cha07 | CHA07 129 | Lyon-15176 | 764 | charcoal | 6845 ± 35 | 7590–7776 | 7607–7744 | 7620–7698 | 7671 |
| Cha07 | CHA07 105 | Lyon-15175 | 618 | charcoal | 6935 ± 35 | 7677–7910 | 7685–7839 | 7710–7825 | 7762 |
| Cha07 | CHA-07 no 7 | Poz-89109 | 472 | charcoal | 7110 ± 40 | 7847–8014 | 7835–7959 | 7856–7939 | 7886 |
| Cha07 | CHA07 79 | Lyon-15174 | 430 | charcoal | 7070 ± 35 | 7797–7968 | 7866–7972 | 7885–7962 | 7923 |
| Cha07 | CHA07 log 3b 45-1 | Poz-127098 | 240 | charcoal | 7560 ± 50 | 8204–8446 | 8205–8445 | 8341–8412 | 8357 |
| Cha07 | CHA-07 no 3 | Poz-89055 | 150 | charcoal | 7190 ± 50 | 7874–8168 | 7931–8158 | 7965–8023 | 7999 |
| Cha07 | CHA07 log1 mal53 | Poz-127097 | 65 | charcoal | 7390 ± 60 | 8035–8342 | 8018–8291 | 8026–8199 | 8117 |
| Cha01 | CHA01 no. 1 | Poz-89054 |  | charcoal | 8840 ± 50 | 9700–10157 |  |  |  |
| Cha08 | CHA08 no2 | Poz-99015 |  | charcoal | 4390 ± 35 | 4855–5231 |  |  |  |
| Bli-IV C57 | BLI C57 ech.1 | DeA-23438 |  | charcoal | 6414 ±40 | 7266 - 7423 |  |  |  |
| Bli-IV C57 | BLI C57 ech.2 | DeA-23439 |  | charcoal | 5597 ±31 | 6302 - 6411 |  |  |  |
| Cha15 | CHA15 log1 ch.3 | Poz-110515 |  | charcoal | 6000 ± 60 | 6675 - 6986 |  |  |  |
| Cha15 | CHA15 n°7 | Lyon-16061 |  | charcoal | 5875 ± 30 | 6631 - 6784 |  |  |  |

Table S1. Radiocarbon dates.

|  | **Taxa** | **Malacological samples** | | | | | | | | | | | | | | | | | | | | | | | | | | |
| --- | --- | --- | --- | --- | --- | --- | --- | --- | --- | --- | --- | --- | --- | --- | --- | --- | --- | --- | --- | --- | --- | --- | --- | --- | --- | --- | --- | --- |
| Groups |  | **M53** | **M51** | **M49** | **M47** | **M45** | **M43** | **M41** | **M39** | **M37** | **M35** | **M33** | **M31** | **M29** | **M27** | **M25** | **M23** | **M21** | **M19** | **M17** | **M15** | **M13** | **M11** | **M9** | **M7** | **M5** | **M3** | **M1** |
| **Aquatic** | *Theodoxus fluviatilis* (Linnaeus, 1758) | 122 | 11 | 82 | 238 | 77 | 208 |  | 16 | 475 | 5 | 8 | 56 | 61 | 208 | 235 | 244 | 367 | 458 | 23 | 158 | 250 | 284 | 130 | 22 | 158 | 163 | 128 |
|  | *Melanopsis* Férussac, 1807 | 200 | 240 | 675 | 8800 | 1045 | 1826 | 15 | 105 | 1414 | 400 | 48 | 385 | 934 | 2758 | 2053 | 3047 | 2093 | 1854 | 397 | 1304 | 1845 | 3288 | 1604 | 238 | 2140 | 2073 | 955 |
|  | *Mercuria* Boeters, 1971 | 175 | 360 | 4105 | 2048 | 1151 | 1050 | 21 | 304 | 2189 | 1640 | 92 | 624 | 192 | 160 | 1217 | 50 | 1080 | 420 | 168 | 530 | 1130 | 3281 | 884 | 206 | 2434 | 632 | 831 |
|  | *Ecrobia* aff. *ventrosa* (Montagu, 1803) |  |  |  |  |  |  |  |  |  |  |  |  |  |  |  |  | 1 |  | 3 | 4 |  | 8 | 4 |  | 20 | 7 |  |
|  | *Radix* sp. | 9 | 1 | 2 | 26 | 7 | 8 |  |  | 4 | 2 |  |  | 1 | 6 | 26 | 4 | 2 |  |  | 18 | 8 | 16 | 10 | 2 | 102 | 31 | 21 |
|  | *Radix auricularia* (Linnaeus, 1758) |  |  |  |  |  |  |  | 1 |  |  |  |  |  |  |  |  |  |  |  |  |  |  |  |  |  |  |  |
|  | *Stagnicola* sp. |  |  | 2 | 4 |  |  |  |  |  |  |  |  |  |  |  |  |  |  |  |  |  |  |  |  |  |  |  |
|  | *Galba truncatula* (Müller, 1774) | 18 | 35 | 742 | 1000 | 112 | 72 | 2 | 28 | 12 | 4 |  | 78 | 2 | 2 | 169 | 4 | 27 | 4 | 6 | 36 | 52 | 132 | 28 | 14 | 134 | 25 | 1 |
|  | *Bulinus truncatus* (Audouin, 1827) |  |  |  |  |  |  |  | 2 |  |  |  |  |  |  |  | 1 |  |  |  | 2 | 4 | 4 |  |  | 140 | 30 | 8 |
|  | *Anisus spirorbis* (Linnaeus, 1758) |  |  |  | 4 | 1 |  |  |  |  |  |  | 2 |  |  |  |  |  |  |  |  |  |  |  |  | 8 | 1 |  |
|  | *Armiger crista* (Linnaeus, 1758) | 235 | 58 | 324 | 254 | 52 | 50 | 7 | 73 | 448 | 456 | 14 | 138 | 53 | 6 | 400 | 1 | 124 | 100 | 22 | 134 | 136 | 528 | 164 | 82 | 698 | 302 | 86 |
|  | *Ancylus fluviatilis* Müller, 1774 | 124 | 90 | 727 | 832 | 113 | 68 | 4 | 57 | 89 | 76 | 16 | 204 | 343 | 26 | 485 | 3 | 1311 | 1560 | 39 | 282 | 280 | 534 | 354 | 176 | 508 | 239 | 52 |
|  | *Potomida littoralis* (Cuvier, 1798) | 1 |  | 1 | 4 | 1 | 2 |  | 1 | 1 |  | 2 | 4 | 3 | 8 | 25 | 3 | 18 | 22 | 6 | 34 | 26 | 28 | 14 | 8 | 30 | 23 | 7 |
|  | *Pisidium casertanum* (Poli, 1791) | 5 | 10 | 120 | 146 | 20 | 4 | 6 | 14 | 29 | 9 | 11 | 30 | 8 | 2 | 4 | 1 | 48 | 28 | 11 | 29 | 24 | 40 | 32 | 16 | 56 | 9 |  |
|  | *Pisidium personatum* Malm, 1855 |  |  |  |  |  |  |  |  |  |  |  |  |  |  |  |  |  |  |  |  |  |  |  |  |  | 2 | 3 |
| **Hygrophile** | *Carychium tridentatum* (Risso, 1826) |  |  | 24 | 16 |  |  |  |  |  |  |  |  |  |  |  |  |  |  | 1 |  |  |  |  |  |  |  |  |
| **Paludal** | Carychium minimum Müller, 1774 | 12 | 58 | 140 | 170 | 22 | 10 | 6 | 19 |  |  | 4 | 20 | 6 |  | 128 | 1 | 20 | 16 | 3 | 22 | 28 | 132 | 54 | 16 | 120 | 23 | 1 |
|  | *Vertigo antivertigo* (Draparnaud, 1801) | 5 | 24 | 196 | 192 | 36 | 50 | 1 | 16 |  | 2 | 1 | 28 | 2 | 4 | 68 | 1 | 12 | 12 | 2 | 28 | 40 | 72 | 20 | 14 | 92 | 33 | 2 |
|  | *Oxyloma elegans (Risso, 1826)* | 9 | 8 | 146 | 204 | 33 | 36 | 1 | 10 | 4 | 2 |  | 20 | 1 | 2 | 46 | 5 | 22 | 28 | 2 | 30 | 28 | 96 | 20 | 6 | 150 | 38 |  |
|  | *Vallonia enniensis* (Gredler, 1856) | 1 | 2 | 73 | 32 | 9 | 8 |  | 2 | 2 |  | 1 | 6 | 6 |  | 48 | 1 | 12 | 8 | 2 | 22 | 20 | 48 | 12 | 4 | 126 | 31 | 5 |
| **Slug** | Slug | 4 |  | 20 |  |  | 2 |  | 1 |  |  |  |  | 2 |  | 4 |  |  |  |  | 2 |  | 12 |  |  | 4 |  |  |
| **Mesophilous** | *Cochlicopa lubrica* (Müller, 1774) | 1 | 1 | 17 |  | 4 | 4 |  | 1 |  |  |  |  |  |  | 16 |  | 8 |  |  |  | 8 | 8 | 2 |  | 14 | 1 | 1 |
|  | *Euconulus fulvus* (Müller, 1774) | 1 |  | 5 | 8 | 3 |  |  | 1 |  |  |  | 2 |  |  |  |  |  |  | 1 |  |  |  |  |  |  |  |  |
|  | *Otala xanthodon* (Anton, 1838) |  |  | 9 | 12 | 9 | 2 |  | 4 | 1 |  |  | 1 |  | 8 | 8 | 4 | 5 |  |  | 12 | 16 | 6 | 6 | 14 | 6 | 12 | 4 |
| **Open area** | *Vertigo pygmaea* (Draparnaud, 1801) |  |  | 8 |  | 1 |  |  |  | 2 |  |  | 2 |  |  | 8 |  | 19 |  |  | 6 |  | 32 | 8 |  | 20 | 5 | 3 |
|  | *Truncatellina* sp. |  |  |  |  |  |  |  |  |  |  |  |  |  |  | 4 |  | 4 |  |  |  |  |  | 2 |  |  |  |  |
|  | *Vallonia pulchella* (Müller, 1774) | 20 | 31 | 521 | 516 | 62 | 52 | 6 | 44 | 16 | 26 | 8 | 104 | 22 | 10 | 412 | 14 | 136 | 76 | 11 | 106 | 184 | 422 | 144 | 52 | 508 | 136 | 15 |
|  | *Vallonia costata* (Müller, 1774) |  |  |  | 12 |  |  |  |  |  |  |  |  |  |  |  |  |  |  |  |  |  | 4 |  |  |  |  |  |
|  | *Pupilla muscorum* (Linnaeus, 1758) |  |  |  |  |  |  |  |  |  |  |  |  |  |  |  |  |  |  |  |  |  |  |  |  |  | 1 |  |
| **Xerothermic** | *Granopupa granum* (Draparnaud, 1801) |  |  |  |  | 1 | 2 |  |  |  |  |  |  |  |  | 1 |  |  |  |  |  |  |  |  |  | 4 | 2 |  |
|  | *Cochlicella barbara* (Linnaeus, 1758) |  |  |  |  |  |  |  |  |  |  |  |  |  |  | 13 |  | 6 |  |  | 2 | 2 | 1 | 2 |  |  |  |  |
|  | *Rumina decollata* (Linnaeus, 1758) |  |  |  | 6 |  | 2 |  |  |  | 1 |  |  |  |  | 4 | 2 |  |  |  |  | 2 | 2 |  |  | 6 | 1 |  |
|  | Geomitridae sp. 1 | 1 | 1 | 9 | 32 | 4 | 6 |  | 1 |  |  |  | 6 | 2 | 4 | 3 | 4 | 7 | 4 |  | 4 | 4 | 14 | 16 |  | 26 | 11 | 5 |
|  | Geomitridae sp. 3 |  |  |  | 2 |  |  |  |  |  |  |  |  |  |  |  |  |  |  |  |  |  |  |  |  |  |  |  |
|  | *Helicopsis* aff. *naini* (Pallary, 1923) |  |  | 1 | 18 | 1 | 2 |  | 1 |  | 4 | 4 | 6 | 5 | 2 | 14 | 6 | 5 | 16 | 5 | 34 | 20 | 54 | 12 |  | 144 | 37 |  |
|  | *Sphincterochila* Ancey, 1887 |  |  |  | 16 |  | 4 |  |  |  |  |  |  | 2 |  |  | 2 |  |  | 3 | 2 | 2 | 2 |  |  |  | 2 |  |
| unclassified taxa | Geomitridae indet. | 7 |  | 36 | 84 | 4 | 20 |  | 4 |  |  |  | 30 | 8 | 8 | 68 | 6 | 44 |  | 4 |  |  |  |  | 10 |  |  |  |
|  | *Ceciliodes acicula* (Müller, 1774) | 1 |  |  | 4 | 1 | 2 |  |  |  |  |  |  | 2 | 2 | 4 |  | 12 | 4 | 1 | 8 | 4 | 4 |  | 4 | 36 | 10 |  |
|  | Total individuals | 951 | 930 | 7985 | 14680 | 2769 | 3490 | 69 | 705 | 4686 | 2627 | 209 | 1746 | 1655 | 3216 | 5463 | 3404 | 5383 | 4610 | 710 | 2809 | 4113 | 9052 | 3522 | 884 | 7684 | 3880 | 2128 |

Table S2. Malacological data of the Cha07 outcrop classified by ecological groups.

| **Depth cm** | **565** |  | **465** |  | **402** |  | **372** |  | **250** |  | **245** |  | **205** |  | **60** |  |
| --- | --- | --- | --- | --- | --- | --- | --- | --- | --- | --- | --- | --- | --- | --- | --- | --- |
|  | % | CV | % | CV | % | CV | % | CV | % | CV | % | CV | % | CV | % | CV |
| **Acer sp** | 0.00 | 0 | 0.00 | 0 | 0.00 | 0 | 0.00 | 0 | 0.00 | 0 | 0.43 | 1 | 0.00 | 0 | 0.00 | 0 |
| **Alnus glutinosa avec arcs** | 0.26 | 1 | 0.00 | 0 | 0.00 | 0 | 0.00 | 0 | 0.00 | 0 | 0.00 | 0 | 0.00 | 0 | 0.00 | 0 |
| **Anthemis sp** | 3.33 | 1 | 0.00 | 0 | 10.81 | 4 | 7.69 | 1 | 0.00 | 0 | 2.63 | 1 | 0.00 | 0 | 0.00 | 0 |
| **Apiaceae** | 0.00 | 0 | 1.03 | 3 | 0.00 | 0 | 0.00 | 0 | 0.30 | 1 | 0.00 | 0 | 0.00 | 0 | 0.27 | 1 |
| **Artemisia sp** | 24.74 | 96 | 3.78 | 11 | 4.47 | 8 | 6.59 | 12 | 13.33 | 44 | 6.93 | 16 | 3.61 | 9 | 2.97 | 11 |
| **Aster t** | 26.67 | 8 | 23.81 | 15 | 16.22 | 6 | 38.46 | 5 | 30.19 | 16 | 18.42 | 7 | 14.29 | 13 | 4.17 | 1 |
| **Atriplex t** | 1.80 | 7 | 17.18 | 50 | 20.11 | 36 | 11.54 | 21 | 1.21 | 4 | 0.43 | 1 | 0.80 | 2 | 1.08 | 4 |
| **Blackstonia t** | 0.00 | 0 | 0.00 | 0 | 0.00 | 0 | 0.00 | 0 | 0.30 | 1 | 0.00 | 0 | 0.00 | 0 | 0.00 | 0 |
| **Cannabis t** | 0.00 | 0 | 0.00 | 0 | 0.00 | 0 | 0.00 | 0 | 0.00 | 0 | 0.43 | 1 | 0.00 | 0 | 0.00 | 0 |
| **Carddus t** | 0.00 | 0 | 0.00 | 0 | 0.00 | 0 | 0.00 | 0 | 0.00 | 0 | 7.89 | 3 | 0.00 | 0 | 0.00 | 0 |
| **Carlina t** | 3.33 | 1 | 3.17 | 2 | 51.35 | 19 | 0.00 | 0 | 1.89 | 1 | 0.00 | 0 | 37.36 | 34 | 8.33 | 2 |
| **Caryophyllaceae** | 0.52 | 2 | 0.00 | 0 | 0.00 | 0 | 1.65 | 3 | 0.00 | 0 | 0.00 | 0 | 0.00 | 0 | 0.27 | 1 |
| **Cedrus sp** | 0.00 | 0 | 0.34 | 1 | 0.00 | 0 | 0.00 | 0 | 0.00 | 0 | 0.43 | 1 | 0.40 | 1 | 0.00 | 0 |
| **Centaurea nigra t** | 0.00 | 0 | 0.00 | 0 | 2.70 | 1 | 0.00 | 0 | 1.89 | 1 | 0.00 | 0 | 0.00 | 0 | 0.00 | 0 |
| **Cerealia 40 µm** | 0.00 | 0 | 0.00 | 0 | 0.00 | 0 | 0.00 | 0 | 0.00 | 0 | 0.87 | 2 | 0.00 | 0 | 0.27 | 1 |
| **Charcoal** |  | 3036 |  | 5202 |  | 168 |  | 54 |  | 436 |  | 437 |  | 0 |  | 400 |
| **Chenopodiaceae** | 0.52 | 2 | 0.00 | 0 | 0.00 | 0 | 1.65 | 3 | 0.00 | 0 | 0.43 | 1 | 0.40 | 1 | 0.00 | 0 |
| **Cichorioideae** | 56.67 | 17 | 53.97 | 34 | 0.00 | 0 | 46.15 | 6 | 49.06 | 26 | 18.42 | 7 | 10.99 | 10 | 50.00 | 12 |
| **Cirsium sp** | 10.00 | 3 | 19.05 | 12 | 18.92 | 7 | 7.69 | 1 | 16.98 | 9 | 52.63 | 20 | 28.57 | 26 | 37.50 | 9 |
| **Corylus sp** | 0.00 | 0 | 0.00 | 0 | 0.00 | 0 | 0.00 | 0 | 0.00 | 0 | 0.00 | 0 | 0.00 | 0 | 0.27 | 1 |
| **Cyperaceae** | 0.00 | 0 | 2.06 | 6 | 0.00 | 0 | 0.55 | 1 | 0.30 | 1 | 1.30 | 3 | 1.20 | 3 | 1.89 | 7 |
| **Cyperus capitatus** | 0.00 | 0 | 0.00 | 0 | 0.56 | 1 | 0.00 | 0 | 0.00 | 0 | 0.00 | 0 | 0.00 | 0 | 0.00 | 0 |
| **Draba t** | 6.44 | 25 | 16.15 | 47 | 21.23 | 38 | 7.14 | 13 | 3.03 | 10 | 2.16 | 5 | 16.06 | 40 | 0.27 | 1 |
| **Echinop sphaerocephalus** | 0.00 | 0 | 0.00 | 0 | 0.00 | 0 | 0.00 | 0 | 0.00 | 0 | 0.00 | 0 | 8.79 | 8 | 0.00 | 0 |
| **Ephedra fragilis t** | 0.00 | 0 | 0.00 | 0 | 0.00 | 0 | 0.00 | 0 | 0.30 | 1 | 1.30 | 3 | 0.40 | 1 | 0.81 | 3 |
| **Euphorbia sp** | 0.77 | 3 | 0.00 | 0 | 0.00 | 0 | 0.00 | 0 | 0.00 | 0 | 0.87 | 2 | 0.40 | 1 | 0.00 | 0 |
| **Geranium sp** | 0.00 | 0 | 0.00 | 0 | 0.00 | 0 | 0.00 | 0 | 3.64 | 12 | 0.00 | 0 | 0.00 | 0 | 0.54 | 2 |
| **Glomus** |  | 90 |  | 0 |  | 1 |  | 5 |  | 1 |  | 5 |  | 0 |  | 0 |
| **Helianthemum sp** | 1.55 | 6 | 0.00 | 0 | 0.56 | 1 | 0.55 | 1 | 0.00 | 0 | 0.00 | 0 | 0.00 | 0 | 0.00 | 0 |
| **Hypericum sp.** | 0.00 | 0 | 0.00 | 0 | 1.12 | 2 | 0.55 | 1 | 0.00 | 0 | 0.43 | 1 | 0.40 | 1 | 0.00 | 0 |
| **Lygeum spartum** | 1.03 | 4 | 0.34 | 1 | 0.00 | 0 | 0.00 | 0 | 0.00 | 0 | 0.43 | 1 | 0.00 | 0 | 0.00 | 0 |
| **Malva sp** | 0.26 | 1 | 0.34 | 1 | 0.00 | 0 | 0.00 | 0 | 0.00 | 0 | 0.00 | 0 | 0.00 | 0 | 0.27 | 1 |
| **Matthiola sp** | 0.26 | 1 | 0.00 | 0 | 0.00 | 0 | 0.00 | 0 | 0.00 | 0 | 0.00 | 0 | 0.00 | 0 | 0.00 | 0 |
| **Mercurialis sp.** | 0.00 | 0 | 0.00 | 0 | 0.56 | 1 | 0.00 | 0 | 0.00 | 0 | 0.00 | 0 | 0.00 | 0 | 0.00 | 0 |
| **Myriophyllum verticilanum** | 0.00 | 0 | 0.00 | 0 | 0.00 | 0 | 0.00 | 0 | 0.00 | 0 | 0.00 | 0 | 0.00 | 0 | 0.27 | 1 |
| **Odontites sp** | 14.43 | 56 | 11.00 | 32 | 2.23 | 4 | 5.49 | 10 | 2.12 | 7 | 2.16 | 5 | 3.21 | 8 | 0.27 | 1 |
| **Olea sp** | 4.64 | 18 | 0.00 | 0 | 3.91 | 7 | 9.89 | 18 | 0.30 | 1 | 0.43 | 1 | 0.40 | 1 | 0.54 | 2 |
| **Ophioglossum lusitanicum sp** |  | 1 |  | 1 |  | 0 |  | 0 |  | 0 |  | 2 |  | 3 |  | 3 |
| **Paronychia sp** | 0.00 | 0 | 0.00 | 0 | 0.00 | 0 | 1.10 | 2 | 0.00 | 0 | 0.00 | 0 | 0.00 | 0 | 0.00 | 0 |
| **Phillyrea sp** | 0.00 | 0 | 0.00 | 0 | 2.23 | 4 | 0.00 | 0 | 0.00 | 0 | 0.00 | 0 | 0.00 | 0 | 0.00 | 0 |
| **Picea sp** | 0.00 | 0 | 0.00 | 0 | 0.00 | 0 | 0.00 | 0 | 0.00 | 0 | 0.43 | 1 | 0.40 | 1 | 0.00 | 0 |
| **Pinus sylvestris** | 2.58 | 10 | 3.78 | 11 | 2.23 | 4 | 7.14 | 13 | 14.24 | 47 | 7.79 | 18 | 4.42 | 11 | 4.59 | 17 |
| **Pinus mediterranean** | 7.22 | 28 | 3.09 | 9 | 6.70 | 12 | 10.99 | 20 | 25.45 | 84 | 17.32 | 40 | 14.46 | 36 | 22.97 | 85 |
| **Pistacia sp** | 0.26 | 1 | 0.00 | 0 | 0.00 | 0 | 0.00 | 0 | 0.00 | 0 | 0.43 | 1 | 0.00 | 0 | 0.00 | 0 |
| **Plantago coronopus** | 4.90 | 19 | 1.03 | 3 | 1.68 | 3 | 7.14 | 13 | 3.33 | 11 | 12.99 | 30 | 1.61 | 4 | 17.30 | 64 |
| **Plantago lanceolata t** | 2.06 | 8 | 0.34 | 1 | 0.00 | 0 | 0.00 | 0 | 0.30 | 1 | 4.76 | 11 | 0.00 | 0 | 0.00 | 0 |
| **Plantago major-media** | 0.77 | 3 | 0.00 | 0 | 0.00 | 0 | 0.00 | 0 | 0.00 | 0 | 0.00 | 0 | 0.80 | 2 | 0.00 | 0 |
| **Poaceae** | 14.69 | 57 | 13.06 | 38 | 2.79 | 5 | 5.49 | 10 | 13.03 | 43 | 10.82 | 25 | 6.43 | 16 | 6.76 | 25 |
| **Podospora sp** |  | 0 |  | 0 |  | 0 |  | 0 |  | 1 |  | 1 |  | 0 |  | 0 |
| **Prunus** | 0.00 | 0 | 0.00 | 0 | 1.12 | 2 | 0.55 | 1 | 0.00 | 0 | 0.43 | 1 | 0.00 | 0 | 0.00 | 0 |
| **Quercus deciduous** | 0.77 | 3 | 1.37 | 4 | 0.56 | 1 | 2.20 | 4 | 0.91 | 3 | 1.73 | 4 | 0.40 | 1 | 1.08 | 4 |
| **Quercus ilex** | 0.00 | 0 | 0.00 | 0 | 0.00 | 0 | 1.10 | 2 | 0.91 | 3 | 0.00 | 0 | 0.00 | 0 | 0.00 | 0 |
| **Reseda sp** | 0.00 | 0 | 0.00 | 0 | 1.12 | 2 | 0.00 | 0 | 0.00 | 0 | 0.43 | 1 | 0.00 | 0 | 0.00 | 0 |
| **Salix sp** | 0.00 | 0 | 0.00 | 0 | 0.00 | 0 | 0.00 | 0 | 0.00 | 0 | 1.30 | 3 | 0.00 | 0 | 0.00 | 0 |
| **Secale** | 0.00 | 0 | 0.00 | 0 | 0.00 | 0 | 0.00 | 0 | 0.00 | 0 | 0.43 | 1 | 0.00 | 0 | 0.00 | 0 |
| **Sedum sp** | 0.00 | 0 | 0.00 | 0 | 1.12 | 2 | 0.00 | 0 | 0.30 | 1 | 0.43 | 1 | 0.80 | 2 | 0.00 | 0 |
| **Sinapis t** | 0.00 | 0 | 0.00 | 0 | 1.12 | 2 | 7.14 | 13 | 0.30 | 1 | 0.43 | 1 | 2.81 | 7 | 0.00 | 0 |
| **Solanum nigrum** | 0.00 | 0 | 0.00 | 0 | 0.56 | 1 | 0.00 | 0 | 0.00 | 0 | 0.00 | 0 | 0.00 | 0 | 0.00 | 0 |
| **Sparganium Typha** | 0.52 | 2 | 3.44 | 10 | 3.35 | 6 | 4.40 | 8 | 0.30 | 1 | 4.33 | 10 | 4.02 | 10 | 30.54 | 113 |
| **Sporormilla sp** |  | 0 |  | 0 |  | 0 |  | 1 |  | 0 |  | 0 |  | 0 |  | 0 |
| **Tamarix** | 1.03 | 4 | 0.00 | 0 | 0.00 | 0 | 0.00 | 0 | 0.00 | 0 | 0.00 | 0 | 0.00 | 0 | 0.00 | 0 |
| **Type 200** |  | 0 |  | 0 |  | 0 |  | 1 |  | 0 |  | 0 |  | 1 |  | 0 |
| **Typha angustifolia** | 0.00 | 0 | 0.00 | 0 | 0.00 | 0 | 0.00 | 0 | 0.00 | 0 | 0.00 | 0 | 0.00 | 0 | 0.27 | 1 |
| **Ulmus sp** | 0.00 | 0 | 0.00 | 0 | 0.00 | 0 | 0.00 | 0 | 0.00 | 0 | 0.43 | 1 | 0.00 | 0 | 0.00 | 0 |
| **Veronica sp.** | 0.26 | 1 | 0.00 | 0 | 0.00 | 0 | 0.00 | 0 | 0.00 | 0 | 0.00 | 0 | 0.00 | 0 | 0.00 | 0 |
| **Pollen sum** |  | 388 | 291 |  | 179 |  | 182 |  | 330 |  | 231 |  | 249 |  | 370 |  |

Table S3. Pollen data of the Cha07 outcrop (CV: counting values).

**Supplementary references**

1. Wengler, L., Vernet, J.L., Michel, P., 1994. Evènements et chronologie de l’Holocène en milieu continental au Maghreb. Les données du Maroc oriental. [Holocene events and chronology in the continental environment of the Maghreb. Data from eastern Morocco]. Quaternaire 5, 119-134.
2. Barathon, J.-J., El Abassi, H., Lechevalier, C., Malek, F., Jolly-Saad, M.-C., 2000. Mise au point sur les formations holocènes dans le Rif oriental (Maroc)/A chronology of Holocene deposits in the eastern Rif mountains (Morocco). Géomorphol. Relief, Process. Environ. 6, 221-238.
3. El Amrani, M., Macaire, J.-J., Zarki, H., Bréhéret, J.-G., Fontugne, M., 2008. Contrasted morphosedimentary activity of the lower Kert River (northeastern Morocco) during the Late Pleistocene and the Holocene. Possible impact of bioclimatic variations and human action. Compt. Rendus Geosci. 340, 533-542.
4. Zielhofer, C., Faust, D., Linstädter, J., 2008. Late Pleistocene and Holocene alluvial archives in the Southwestern Mediterranean: Changes in fluvial dynamics and past human response. Quaternary International 181, 39–54.
5. Bartz, M., Rixhon, G., Kehl, M., El Ouahabi, M., Klasen, N., Brill, D., Weniger, G.-C., Mikdad, A., Brückner, H., 2017. Unravelling fluvial deposition and pedogenesis in ephemeral stream deposits in the vicinity of the prehistoric rock shelter of Ifri n’Ammar (NE Morocco) during the last 100 ka. Catena 152, 115–134.
6. Ibouhouten, H., Zielhofer, C., Mahjoubi, R., Kamel, S., Linstädter, J., Mikdad, A., Bussmann, J., Werner, P., Härtling, J.W., Fenech, K., 2010. Archives alluviales holocènes et occupation humaine en Basse Moulouya (Maroc nord-oriental). Géomorphologie : relief, processus, environnement 16, 41–56.
7. Zielhofer, C., Bussmann, J., Ibouhouten, H., Fenech, K., 2010. Flood frequencies reveal Holocene rapid climate changes (Lower Moulouya River, northeastern Morocco). Journal of Quaternary Science 25, 700–714.
8. Walker, M., Head, M.J., Berkelhammer, M., Björck, S., Cheng, H., Cwynar, L., Fisher, D., Gkinis, V., Long, A., Lowe, J., Newnham, R., Rasmussen, S.O., Weiss, H., 2018. Formal ratification of the subdivision of the Holocene Series/Epoch (Quaternary System/Period): two new Global Boundary Stratotype Sections and Points (GSSPs) and three new stages/subseries. Episodes 41, 213–223.
9. Puisségur, J. J., 1976. Mollusques continentaux quaternaires de Bourgogne. (Mémoires géologiques de l’université).
10. Wengler L. and Vernet J.L., 1992. Vegetation, sedimentary deposits and climates during the late Pleistocene and Holocene in eastern Morocco. Palaeogeography, Palaeoclimatology, palaeoecology, 94, 141-167.
11. Triat-Laval H., 1978. - Contribution pollenanalytique à l'histoire tardiglaciaire et postglaciaire de la végétation de la basse vallée du Rhône. Thèse, Aix- Marseille III, 343 p.
12. Benslama, M., Andrieu-Ponel, V., Guiter, F., Reille, M., Beaulieu (de), J.-L., Migliore, J., Djamali, M., 2010. Contribution à l’histoire holocène de la végétation en Algérie : analyses polliniques de deux profils sédimentaires du complexe humide d’El-Kala. Comptes rendus de l’Académie des Sciences série Biologie, 333, 744-754.
